# Air temperature and all-cause emergency hospital admissions in people with and without diabetes in Germany (2005–2022): a time-series analysis

**DOI:** 10.1016/j.lanepe.2026.101591

**Published:** 2026-01-23

**Authors:** Thaddäus Tönnies, Marielle Wirth, Katharina Piedboeuf-Potyka, Oliver Kuss

**Affiliations:** a Institute for Biometrics and Epidemiology, German Diabetes Center, Leibniz Center for Diabetes Research at the Heinrich Heine University Düsseldorf, Düsseldorf, Nordrhein-Westfalen, Germany; b Centre for Health and Society, Medical Faculty and University Hospital Düsseldorf, Heinrich Heine University Düsseldorf, Düsseldorf, Germany; c German Center for Diabetes Research, München-Neuherberg, Germany

**Keywords:** Diabetes, Heat, Cold, Morbidity, Hospitalization, Climate change

## Abstract

**Background:**

People with diabetes may be more vulnerable to temperature extremes due to impaired thermoregulation and higher prevalence of comorbidities, but evidence is limited. We aimed to compare short-term effects of extreme heat and cold on all-cause emergency hospital admissions in Germany among people with and without diabetes.

**Methods:**

We applied space- and time-stratified conditional quasi-Poisson regression with distributed lag non-linear models (up to 21 days) to estimate short-term effects of daily average temperature. We chose the reference temperature (20 °C) such that it approximates the minimum morbidity temperature in most subgroups. Analyses were stratified by sex, age, and diabetes status using data from all emergency hospital admissions in Germany, 2005–2022 (N = 132,243,083) at the level of 400 administrative districts, enabling an ecological study.

**Findings:**

Both heat and cold increased hospital admissions. Heat-related relative risks (RR) were broadly similar between people with and without diabetes. Considering all ages, heat-related RRs (95% confidence interval) were 1.03 (1.03–1.04), 1.07 (1.00–1.13), and 1.02 (1.01–1.03) in males without, with type 1, and with type 2 diabetes. Age-specific RRs for heat and cold were similar between people without and with type 2 diabetes but higher for type 1 diabetes in some subgroups; e.g. cold-related RRs were 1.13 (1.12–1.15) and 1.51 (1.14–2.01) in men aged ≥80 years without and with type 1 diabetes.

**Interpretation:**

Contrary to prior hypotheses, diabetes was not associated with greater vulnerability. This may reflect good healthcare access and increased awareness of heat and cold-related risks among people with diabetes. Nevertheless, given the higher baseline risk of hospital admission in diabetes, similar RR may still translate into larger absolute effects of extreme temperatures. Hence, clinical practice and policies aimed at mitigating temperature-related effects should continue to consider diabetes as a potential vulnerability factor.

**Funding:**

None.

**Research in context**

**Evidence before this study**

We searched PubMed and Google Scholar from inception until 29 Aug 2025 for peer-reviewed papers on short–term associations between air temperature and diabetes mortality and morbidity with a particular focus on hospital admissions. We used the search terms ∗air temperature∗ OR ∗heat∗ OR ∗cold∗ AND ∗mortality∗ OR ∗morbidity∗ OR ∗hospitalization∗ AND ∗diabetes∗. We excluded studies not related to diabetes and focused on evidence from systematic reviews and meta-analyses, if available. Evidence from meta-analyses indicates that temperature extremes increase both diabetes-related mortality and morbidity. Moreover, individual studies suggest an increased risk for acute metabolic complications such as hyperglycaemia and hypoglycaemia. However, few studies on diabetes-related outcomes have been conducted in Europe, despite the region's higher-than-average rates of temperature-related mortality and unique healthcare contexts. Most existing studies focused on cause-specific hospital admissions for diabetes but did not compare effects between people with and without diabetes. Additionally, little is known about whether type 1 and type 2 diabetes are affected differently, despite their distinct underlying pathophysiology.

**Added value of this study**

This study provides new insights into the short-term effects of air temperature on emergency hospital admissions in Germany, with a specific focus on differences between people with and without diabetes. This is the first study that assessed all-cause admissions and directly compared temperature-related risks between people with type 1 diabetes, type 2 diabetes, and no diabetes, while also examining variations by age and sex. Using nationwide data covering the entire German population, we found that vulnerability to temperature extremes is strongly age-dependent, with older adults generally at higher risk. Contrary to our expectations, type 2 diabetes was not associated with greater heat vulnerability, while people with type 1 diabetes tended to be more susceptible, but estimates were too imprecise for definitive conclusions. Overall, this study expands current knowledge by identifying heterogeneity in temperature effects by age, sex and diabetes type.

**Implications of all the available evidence**

Together, existing evidence and our findings show that temperature extremes generally increase the risk of hospital admissions. However, it remains uncertain whether diabetes consistently modifies heat or cold-related effects. Regional differences in climate, healthcare, and population health likely influence the associations, underlining the need for context-specific research. Future work should compare people with and without diabetes across a broader set of cause-specific hospital admissions to provide a more comprehensive picture of health impacts. Furthermore, the potential impact of diabetes-related comorbidities, such as chronic kidney disease, on temperature effects requires closer examination.

## Introduction

Ambient air temperature is associated with short-term mortality and morbidity.^1^, ^2^, ^3^, ^4^, ^5^ People with diabetes are hypothesized to be particularly vulnerable to the short-term effects of extreme heat.^6^, ^7^, ^8^ This vulnerability may be due to impaired thermoregulation^9^ and increased cardiovascular risk profiles,^10^ which together reduce the capacity to cope with extreme temperatures. This is supported by systematic reviews and meta-analyses,^11^^,^^12^ which for example found that diabetes mortality increased by 18% (95%-confidence interval: 13–25%) during heat waves.^12^ Diabetes morbidity in terms of hospital admissions, emergency department visits and outpatient visits were increased by 10% (6–14%).^12^ One Portuguese study found that heatwaves affected hospital admissions differently across the 25 main disease categories defined by the International Classification of Diseases (ICD).^13^ Admissions for endocrine, nutritional, and metabolic disorders ranked fourth on the list, showing a 25% (23–27%) increase during periods of extreme heat. The authors explained this rise with the impaired capacity of people with diabetes to adapt to high temperatures. A Japanese study found a 64% (38–93%) increased risk for hyperglycaemia hospital admissions at the 99th percentile vs. the 75th percentile of the daily temperature distribution.^14^ Likewise, the risk for hypoglycaemia hospital admissions was increased by 65% (29–110%). In addition to heat, cold temperatures are also associated with increased morbidity and mortality, particularly in regions with temperate climates where cold-related effects often exceed those of heat.^4^ Moreover, there is evidence that diabetes mortality and morbidity are particularly increased during episodes of extreme cold.^15^

Despite this evidence, the current body of literature on potentially increased vulnerability among people with diabetes faces substantial research gaps. First, there are very few studies from Europe investigating the effect of air temperature on diabetes-related hospital admissions. For instance, in a systematic review, only one of 15 included study populations was from Europe and none from Germany.^12^ This is problematic, as the impact of temperature on morbidity and mortality likely depends, among other things, on regional climatic conditions, the overall health status of the population and access to outpatient healthcare. Moreover, Europe was found to experience higher than global average rates of both cold- and heat-related mortality,^2^ suggesting that people with diabetes in Europe may be particularly vulnerable to temperature-related health consequences. Second, most studies considered morbidity in terms of cause-specific outcomes (e.g. diabetes as primary diagnosis for hospital admissions). Although this approach provides important insights, it excludes other causes of temperature-related morbidity that may be increased among people with diabetes (e.g. hospital admissions due to cardiovascular diseases). Third, there are only very few studies that differentiated temperature effects by diabetes type. Given the distinct pathophysiology of type 1 and type 2 diabetes, with type 1 diabetes characterized by absolute insulin deficiency and type 2 diabetes by insulin resistance and β-cell dysfunction, it seems likely that temperature effects differ between these groups. For instance, there are substantial differences in the cardiovascular risk profile between these diabetes types^10^ and cardiovascular outcomes are particularly important for heat-related risks.^16^

In the context of climate change, these research gaps are becoming increasingly relevant, because heat stress is projected to rise substantially in the future, particularly in Europe.^17^ Moreover, the prevalence of diabetes has been increasing for decades, with no indication that this trend is about to stop.^18^ The potentially increased vulnerability to temperature extremes among people with diabetes could therefore become a major driver of future temperature-related mortality and morbidity.

To address these issues, we aimed to estimate the short-term effects of air temperature on the risk of emergency hospital admission due to all causes in Germany, and to compare effects between people with and without diabetes by diabetes type, age and sex.

## Methods

### Study population and data sources

In this ecological study, we used daily data from all hospital admissions in Germany between 2005 and 2022 from the Diagnosis Related Groups (DRG) Statistic.^19^ These data are documented for reimbursement in the German DRG system and include information on diagnoses, procedures, demography, date of admission and place of residence of the patients. Diagnoses are coded according to ICD-10 and include one primary and up to 89 secondary diagnoses. All diagnoses, together with documented procedures (e.g. surgeries, diagnostics), are used to assign the final DRG category, which directly determines the reimbursement amount the hospital receives for that case. We included all age groups and all causes for hospital admissions, but restricted the analysis to emergency hospital admissions, assuming that temperature does not affect scheduled hospital admissions. Admissions of patients with a recorded place of residence outside Germany, unknown residence or missing information on sex or age were excluded from all analyses. We transformed the individual level DRG data to multi-location time series data with the daily number of hospital admission per German administrative district (‘Kreise und kreisfreie Städte’, N = 400) by subgroups of diabetes status, sex and age.

We linked the time-series DRG data to daily mean temperature from the ENSEMBLES Observations 0.1° × 0.1° gridded dataset (E-OBS). Linkage was achieved by date of admission and place of residence on the district level. E-OBS is based on interpolated observations from a dense network of 25,000 weather stations and has been widely used in epidemiological studies, ensuring comparability across European regions. It is freely available from Copernicus Climate Change Service.^20^^,^^21^

### Variables

People with diabetes were identified by ICD-10 codes E10 to E14 documented as primary or secondary diagnoses. The distinction between type 1 diabetes and type 2 diabetes followed definitions of the German diabetes surveillance.^22^ Modifications were necessary, mainly because in contrast to Reitzle et al.,^22^ we had no information on insulin and oral antidiabetic drugs use. Briefly, type 1 diabetes was assigned to individuals with an E10 diagnosis. Type 2 diabetes was defined by the presence of E11, E12, or E14 diagnoses. People with both E10 (type 1 diabetes) and E11 diagnoses (type 2 diabetes) were categorized as having type 1 diabetes if E10 was the primary diagnosis or age was <20 years. All other cases with documented E10 and E11 were categorized as type 2 diabetes. Individuals with E13 diagnosis only or with combinations of E13 and E14 were classified as ‘other diabetes’ and excluded from further analysis. People without ICD-10 codes E10 to E14 were grouped into ‘no diabetes’.

Further variables considered during the analysis were sex with categories ‘male’ and ‘female’ as well as age groups <50 years, 50–59 years, 60–69 years, 70–79 years and ≥80 years. Place of residence was available on the level of the 400 German districts.

E-OBS daily temperature data is provided on a 0.1° × 0.1° grid. In E-OBS, the daily mean temperature is derived as the arithmetic mean of the daily maximum and minimum temperature. We spatially aggregated these gridded data to the level of 400 German administrative districts by calculating the area-weighted mean for each day and district. Grid cells that intersected with more than one district were included in the calculation, with their contribution weighted proportionally to the area of the cell falling within each respective district. The daily number of hospital admissions in subgroups of sex, diabetes status and age was linked to daily mean temperature by district and date of admission such that each subgroup in each district received the corresponding daily temperature. Lagged temperature values up to lag day 21 were added to account for delayed effects of air temperature in the analysis.

### Statistical analysis

Data preparation and description were carried out in SAS (SAS Institute Inc., Cary, NC). All other analyses were performed with R (R Foundation for Statistical Computing, Vienna, Austria).

To estimate the short-term effect of daily air temperature on the risk of hospital admission, we used space and time-stratified conditional quasi-Poisson regression with the daily number of hospital admissions as the dependent variable. We stratified the model by district, year and month. This model is equivalent to a model with an intercept for each district and time stratum, without explicitly estimating the intercepts.^23^ By this, the model estimates the effects within strata of a given year, month and district, thereby adjusting for potential confounding due to regional differences, long-term temporal and seasonal trends.^23^ Additionally, we adjusted for a potential weekday effect, by including the day of the week as an independent categorical variable in the regression model.

The temperature effect was estimated with distributed lag non-linear models (DLNM).^24^ These models allow to simultaneously model the non-linear dose–response association and the non-linear lag-response association. By this, DLNM account for immediate and potentially lagged effects of temperature. As in previous studies,^25^ we fitted a natural cubic spline to model the dose–response association, placing knots at the 10th, 75th, and 90th percentiles of the temperature distribution. For the lag-response association, we used a natural cubic spline with three equally spaced knots on the log scale, covering lag days 0–21.

To investigate whether the effects differ by diabetes status, we fitted separate models for people without diabetes, with type 1 diabetes and type 2 diabetes within subgroups of age and sex. We report results in terms of overall cumulative dose–response associations, which summarize the temperature effect over all lag days by aggregating lag-specific dose–response associations.^24^ To facilitate interpretation, U-shaped temperature-morbidity associations are usually reported relative to the minimum morbidity temperature (MMT). In our study, some subgroups did not exhibit clear U-shaped relationships. Therefore, we initially used the mean of observed daily temperatures (9.8 °C) as the reference for predicting relative risks (RRs) from the DLNM. For interpretability, we subsequently rescaled the RRs and CIs to a reference of 20 °C by dividing all RRs and CI-limits by the RR observed at 20 °C. We chose 20 °C as reference because in subgroups with U-shaped relations, 20 °C was generally close to the MMT. Centring at subgroup-specific MMTs would hinder comparability between subgroups, as the RRs would then refer to different reference temperatures.

We conducted several additional analyses to assess consistency with previous studies and the robustness of our results. These included restricting analyses to non-external causes of admission (ICD-10 A00–R99), stratifying by urbanicity (rural, rural-urban, urban), restricting to cause-specific diabetes hospitalizations (diabetes as the primary diagnosis), using ten lag days instead of 21, and restricting the analysis to the pre-pandemic period (2005–2019).

### Ethics statement

For this study, only secondary data documented for reimbursement purposes were used without any collection of additional data on individuals. Due to privacy protection rules, access to these data were only possible indirectly and in anonymized and highly aggregated form. The use of such anonymized secondary data is not subject to ethics committee approval and informed consent in Germany.

### Role of the funding source

The German Diabetes Center is funded by the German Federal Ministry of Health and the Ministry of Culture and Science of the state of North Rhine-Westphalia. The German Center for Diabetes Research is funded by the German Federal Ministry of Education and Research. Neither of these funders had any role in the design, execution, analyses, interpretation of data or decision to submit results in this study. In particular, the research reported here received no specific grant from any funding agency in the public, commercial or not-for-profit sectors.

## Results

**Fig. 1:**
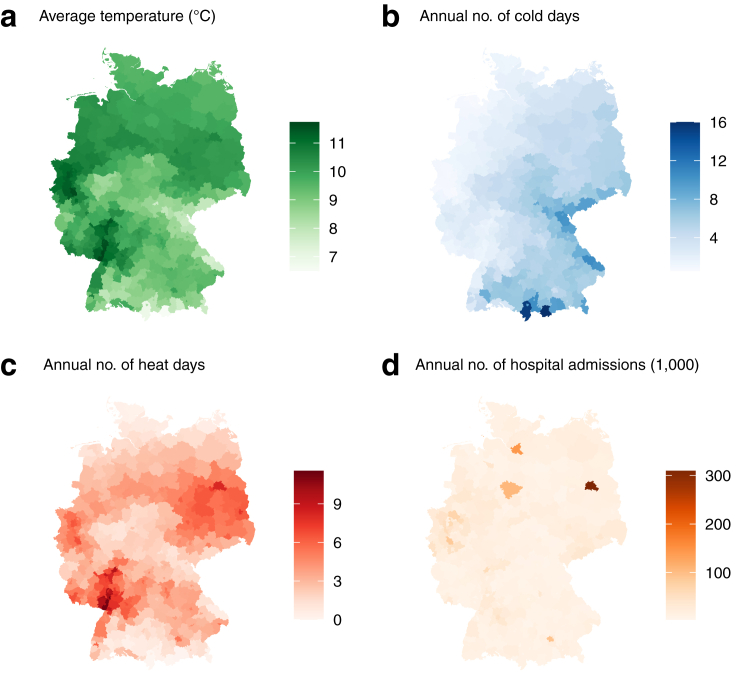
**Spatial distribution of air temperature and emergency hospital admissions in Germany between 2005 and 2022**. The different maps show the long term average daily temperature (a), average annual number of cold (b) and heat (c) days and the average annual number of hospital admissions (d). Thresholds for heat and cold days were the 1st (−6.5 °C) and 99th (24.5 °C) percentile of the overall temperature distribution.

After exclusion of 410,413 (0.3%) observations with unclear diabetes status, the final analysis data set comprised 132 million emergency hospital admissions (Table 1). The proportion of males was larger among people with type 2 diabetes but similar with regard to type 1 diabetes. With increasing age the frequency of type 2 diabetes increased whereas it decreased for type 1 diabetes. This pattern reflects the age distribution of diabetes incidence with type 1 peaking in childhood and adolescence, while type 2 develops later and peaks around age 75 years. With age, type 1 becomes less frequent due to excess mortality from long disease duration and complications. The number of emergency admissions with type 2 diabetes increased from 709,057 in 2005 to 1,341,225 in 2022. Fig. 1 shows the spatial distribution of air temperature and emergency hospital admissions in 400 administrative districts.

**Table 1:** Description of all emergency hospital admissions in Germany between 2005 and 2022.

	No diabetes	Type 1 diabetes	Type 2 diabetes	Total
N	110,085,054 (100.0%)	736,408 (100.0%)	21,421,621 (100.0%)	132,243,083 (100.0%)
Sex				
Male	50,974,322 (46.3%)	393,502 (53.4%)	10,869,189 (50.7%)	62,237,013 (47.1%)
Female	59,110,732 (53.7%)	342,906 (46.6%)	10,552,432 (49.3%)	70,006,070 (52.9%)
Age (years)				
<50	43,399,341 (39.4%)	414,250 (56.3%)	778,525 (3.6%)	44,592,116 (33.7%)
50–59	12,564,014 (11.4%)	101,274 (13.8%)	1,828,090 (8.5%)	14,493,378 (11.0%)
60–69	14,051,764 (12.8%)	84,568 (11.5%)	3,922,469 (18.3%)	18,058,801 (13.7%)
70–79	18,353,109 (16.7%)	81,039 (11.0%)	7,149,482 (33.4%)	25,583,630 (19.3%)
>79	21,716,826 (19.7%)	55,277 (7.5%)	7,743,055 (36.1%)	29,515,158 (22.3%)
Year				
2005	4,597,849 (4.2%)	36,871 (5.0%)	709,057 (3.3%)	5,343,777 (4.0%)
2006	4,839,452 (4.4%)	37,089 (5.0%)	770,907 (3.6%)	5,647,448 (4.3%)
2007	5,045,096 (4.6%)	36,509 (5.0%)	838,511 (3.9%)	5,920,116 (4.5%)
2008	5,295,942 (4.8%)	37,524 (5.1%)	907,578 (4.2%)	6,241,044 (4.7%)
2009	5,533,567 (5.0%)	36,056 (4.9%)	984,835 (4.6%)	6,554,458 (5.0%)
2010	5,684,336 (5.2%)	35,961 (4.9%)	1,051,113 (4.9%)	6,771,410 (5.1%)
2011	5,932,565 (5.4%)	36,537 (5.0%)	1,124,080 (5.2%)	7,093,182 (5.4%)
2012	6,195,285 (5.6%)	37,302 (5.1%)	1,193,422 (5.6%)	7,426,009 (5.6%)
2013	6,428,391 (5.8%)	38,838 (5.3%)	1,261,390 (5.9%)	7,728,619 (5.8%)
2014	6,671,421 (6.1%)	39,986 (5.4%)	1,327,279 (6.2%)	8,038,686 (6.1%)
2015	6,871,602 (6.2%)	42,618 (5.8%)	1,388,232 (6.5%)	8,302,452 (6.3%)
2016	7,065,444 (6.4%)	44,508 (6.0%)	1,422,919 (6.6%)	8,532,871 (6.5%)
2017	7,073,441 (6.4%)	45,027 (6.1%)	1,452,520 (6.8%)	8,570,988 (6.5%)
2018	7,058,660 (6.4%)	47,153 (6.4%)	1,456,142 (6.8%)	8,561,955 (6.5%)
2019	7,116,813 (6.5%)	48,081 (6.5%)	1,479,663 (6.9%)	8,644,557 (6.5%)
2020	6,200,977 (5.6%)	43,448 (5.9%)	1,352,413 (6.3%)	7,596,838 (5.7%)
2021	6,231,697 (5.7%)	45,755 (6.2%)	1,360,335 (6.4%)	7,637,787 (5.8%)
2022	6,242,516 (5.7%)	47,145 (6.4%)	1,341,225 (6.3%)	7,630,886 (5.8%)

Data are shown as n (%).

**Fig. 2:**
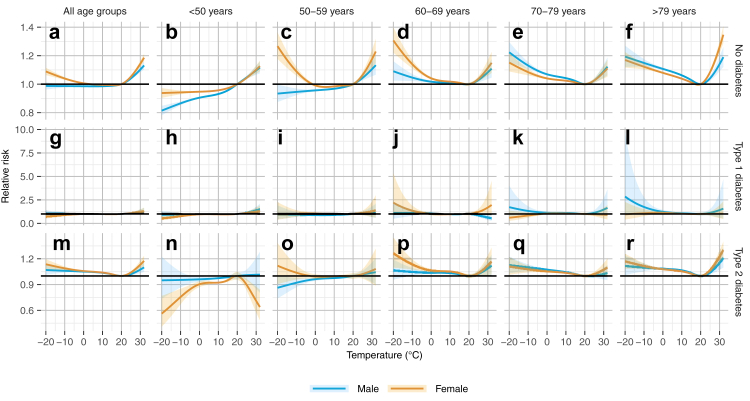
**Overall cumulative association between daily air temperature and all-cause emergency hospital admission by age, sex and diabetes status**. The panels show results for people without diabetes (a–f), with type 1 diabetes (g–l), type 2 diabetes (m–r) including all age groups (a, g, m), age <50 years (b, h, n), 50–59 years (c, i, o), 60–69 years (d, j, p), 70–79 years (e, k, q) and >79 years (f, l, r). Estimates are based on separate conditional quasi-Poisson regressions with distributed lag non-linear models including lag days 0–21.

Fig. 2 shows overall cumulative temperature-morbidity associations from DLNMs by age, sex and diabetes status with 20 °C as the reference temperature. The results indicate substantial heterogeneity. Considering all age groups combined, there was a U-shaped relation among people with type 2 diabetes for both, males and females, whereas among people without diabetes, this was only the case for females. Age-specific associations show that the U-shape is mainly driven by age groups ≥60 years. In contrast, for age groups <60 years, the results partly indicate a protective association for cold temperatures, in particular among males. The increase in risk due to warmer and colder temperatures tended to be larger with increasing age, although this pattern was not consistent. For women without diabetes aged between 50 and 69 years, the risk increase due to cold temperatures was considerably larger than among men.

**Fig. 3:**
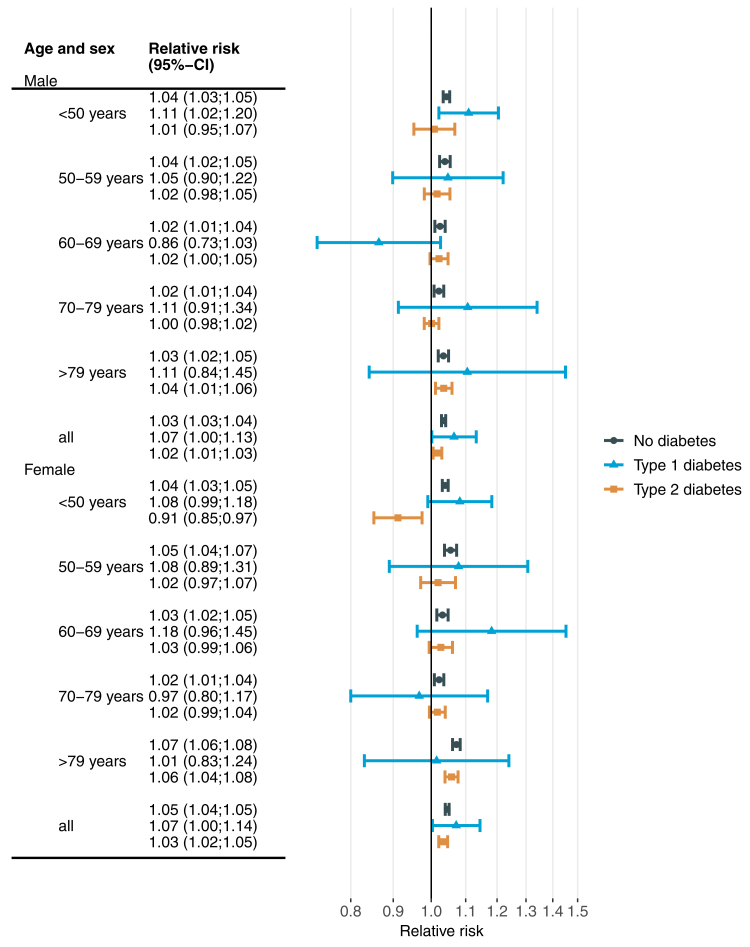
**Overall cumulative association between extreme heat and all-cause emergency hospital admission by age, sex and diabetes status**. Extreme heat was defined as daily average temperature of 24.5 °C (99th percentile of the daily temperature distribution) and compared to a reference temperature of 20 °C. Estimates are based on separate conditional quasi-Poisson regressions with distributed lag non-linear models including lag days 0–21.

**Fig. 4:**
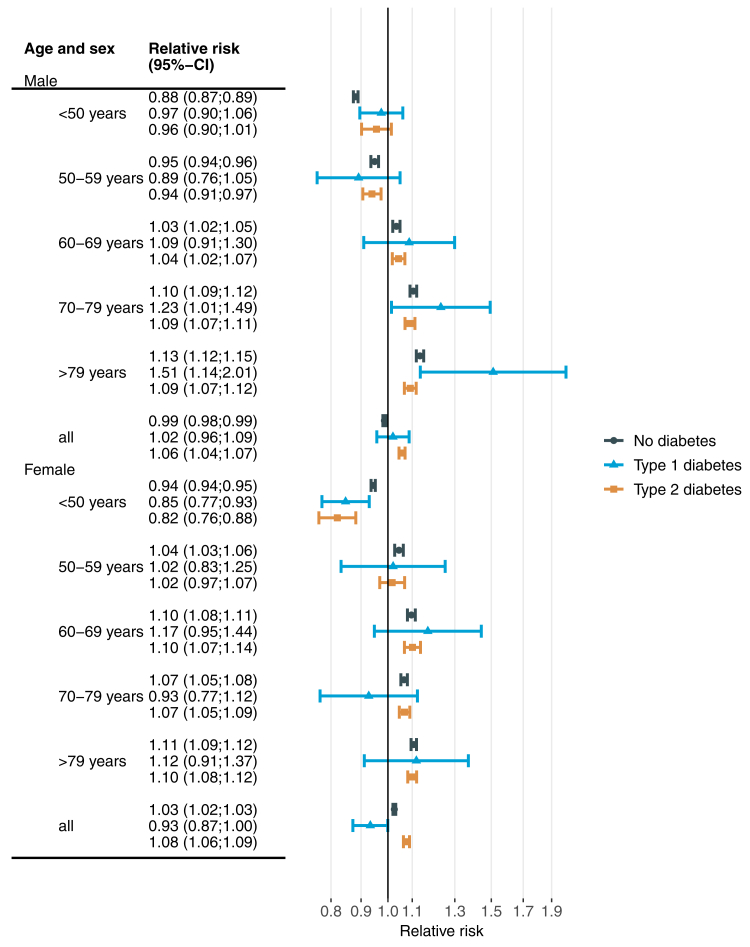
**Overall cumulative association between extreme cold and all-cause emergency hospital admission by age, sex and diabetes status**. Extreme cold was defined as daily average temperature of −6.5 °C (1st percentile of the daily temperature distribution) and compared to a reference temperature of 20 °C. Estimates are based on separate conditional quasi-Poisson regressions with distributed lag non-linear models including lag days 0–21.

To compare the effects of extreme heat and cold between people with and without diabetes, Fig. 3 shows RRs associated with the 99th percentile (24.5 °C) of the observed daily mean temperature and Fig. 4 shows corresponding estimates for the 1st percentile (−6.5 °C). For all age groups combined, RRs (95%-confidence interval) related to extreme heat were 1.03 (1.03–1.04), 1.07 (1.00–1.13) and 1.02 (1.01–1.03) for males without diabetes, with type 1 diabetes and type 2 diabetes, respectively. Corresponding estimates for females were 1.05 (1.04–1.05), 1.07 (1.00–1.14) and 1.03 (1.02–1.05) (Fig. 3). The RRs for extreme heat were highest for type 1 diabetes in most age groups among males, and generally slightly lower for people with type 2 diabetes compared to people without diabetes. The latter observation also holds for females.

For all age groups combined, RRs for extreme cold (Fig. 4) were 0.99 (0.98–0.99), 1.02 (0.96–1.09) and 1.06 (1.04–1.07) for males without diabetes, with type 1 diabetes and type 2 diabetes, respectively. Corresponding estimates for females were 1.03 (1.02–1.03), 0.93 (0.87–1.00) and 1.08 (1.06–1.09). Among males, the RR for extreme cold increased with increasing age, ranging from a protective association of 0.89 (0.76–1.05) in age group 50–59 years (type 1 diabetes) to a harmful association of 1.51 (1.14–2.01) in age group ≥80 years (type 1 diabetes). This trend was less pronounced among females. For women without diabetes aged between 50 and 69 years, the risk increase due to cold temperatures was considerably larger than among men. Extreme cold tended to be larger than extreme heat effects, e.g. in age group ≥80 years.

Corresponding lag-response associations for heat (Figure S1) and cold (Figure S2) show expected patterns with more immediate effects for heat and prolonged effects for cold.

Considering the uncertainty in the estimates, the results do not show a clear trend toward either higher or lower RR for individuals with diabetes compared to those without, for both extreme heat and extreme cold conditions.

Details of the additional analyses are provided in the Supplementary Material. No substantial differences to the main analysis were observed when restricting analyses to the pre-pandemic period (Figures S3–S5), or further stratifying by urbanicity (Figures S6–S14). Considering ten instead of 21 lag days did not change heat-related RR but attenuated cold-related RR, possibly indicating that the prolonged cold effect is not adequately captured by the shorter lag period (Figures S15–S17). Likewise, restricting to non-external causes did not change heat-related RR but attenuated cold-related RR (Figures S18–S20). Restricting the analysis to cause-specific diabetes hospital admissions showed slightly higher heat-related RR for type 2 diabetes whereas RR for cold were attenuated (Figures S21–S23). Overall, none of the additional analyses changed the results meaningfully, in particular regarding differences in RR between people with and without diabetes.

## Discussion

This study is the first to assess short-term effects of air temperature on all-cause emergency hospital admissions in people without diabetes, with type 1 and type 2 diabetes. We observed substantial heterogeneity in heat and cold effects, driven mainly by age, with smaller differences by sex and diabetes status. Extreme heat was associated with increased admission risk in most subgroups, particularly among those aged >60 years, whereas diabetes and sex had smaller impacts on effect estimates. Contrary to prior hypotheses, type 2 diabetes was not associated with greater heat vulnerability compared with no diabetes. There is some suggestion that people with type 1 diabetes may be more affected by heat than those with type 2 diabetes or without diabetes, but the uncertainty is large, mainly due to smaller sample sizes for type 1 diabetes. Extreme cold was associated with reduced risk in some groups aged <60 years and increased risk in most groups aged ≥60 years. The largest cold-related risk occurred in men aged ≥80 years with type 1 diabetes. In other groups, there was little indication that either diabetes type had greater cold-related vulnerability than those without diabetes.

One previous study using the same data concluded that both extreme heat and cold are associated with increased morbidity, but did not stratify the analyses by, age, sex or diabetes status.^26^ We are not aware of studies reporting protective effects of colder temperatures on all-cause hospital admissions in younger age groups. This finding may be explained in relation to hospital admissions due to external causes. These causes are frequent in adolescents and young adults whereas causes typical for older ages (e.g. cardiovascular) are less common. Extremely cold temperatures might decrease risky behaviour in younger age groups leading to fewer accidents, resulting in protective effects of colder temperatures on all-cause hospital admissions. For instance, one Japanese study found the lowest rate of transport-related deaths, typical for people in younger age, during cold temperatures and a roughly positive linear relation with air temperature. In contrast, deaths due to falls, typical for older age groups, were increased during hot and cold temperatures.^27^ One Chinese study found an approximately negative linear relation between hospital admissions due to injuries with lower risk in colder temperatures.^28^ On the other hand, one systematic review found that cold temperatures were associated with increased cardiovascular mortality also among people aged <65 years.^29^

One previous study compared temperature effects on hospital admissions due to acute myocardial infarction (AMI) between people with and without diabetes (all types combined). Contrary to our results, the authors found that people with diabetes are more strongly affected by extreme heat and cold.^30^ There are several potential reasons for these disagreeing findings. First, we included all causes for hospital admission and not only those due to AMI. It seems possible that heat and cold effects on specific causes for hospital admission are larger among people with diabetes without necessarily translating to differences in all-cause hospital admissions. Moreover, differences in access to outpatient care and climatic conditions between Germany and Hong Kong could explain different findings. One methodological issue could also explain the findings of Lam et al.^30^ They used two broad age groups with a cut-off at 75 years. These large age groups can only partly account for age differences in people with vs. without diabetes. For instance people with diabetes in the age group <75 years are presumably older on average than people without diabetes in the same age group. Hence, increased heat and cold effects among people with diabetes may partly be explained by remaining differences in the age distribution. The fact that the differences in effect size between people with vs. without diabetes in Lam et al.^30^ were more pronounced in the much larger <75 years age group than in the smaller ≥75 years age group supports this reasoning. Our study is less prone to this potential bias, since we analysed the data in six smaller age groups. In addition, two other studies from Norway and Belgium are in line with our findings suggesting no increased vulnerability in people with diabetes regarding heat and cold effects on natural cause mortality.^31^^,^^32^

Our findings contrast with previous evidence suggesting that heat increases cause-specific diabetes morbidity^11^, ^12^, ^13^^,^^33^ whereas we found no increased vulnerability regarding all-cause hospital admissions. Although previous studies focused on hospital admissions due to diabetes and not on all-cause admissions in people with diabetes, this is a rather surprising finding. One might reasonably expect that heat-related increases in diabetes hospital admissions, together with the wide range of diabetes-associated comorbidities affected by heat (e.g. cardiovascular disease, kidney disease), would also translate into an elevated vulnerability in all-cause hospital admissions. We found only weak evidence that people with type 1 diabetes may be more vulnerable to heat exposure compared to people without diabetes. However, due to the comparatively small number of hospital admissions among individuals with type 1 diabetes, statistical uncertainty remains considerable.

When comparing temperature effects across subgroups, one important methodological issue should be noted. We examined modification of the temperature effect by diabetes only on a relative scale by estimating RRs per subgroup. Similar RRs between individuals with and without diabetes may nonetheless correspond to different absolute risk differences, if baseline risks at the reference temperature differ. This distinction is critical, for example, when considering temperature-related excess morbidity and mortality. Potentially larger absolute effects observed among people with diabetes translate into a larger number of emergency hospital admissions at the population level. For example, in 2015, there were about 6.8 million people with type 2 diabetes in Germany in a population of 82.2 million.^34^ With 1.4 million hospital admissions with type 2 diabetes and 6.9 million without diabetes in 2015 (Table 1), average daily admission rates were 55.9 and 25.0 per 100,000 people, respectively. Applying heat-related RRs of 1.03 for type 2 diabetes and 1.05 for no diabetes (Fig. 3, females, all age groups), the rates increase to 57.6 and 26.2 per 100,000. In this rough approximation, the absolute risk increase due to heat is higher for people with type 2 diabetes (1.7 additional admissions per 100,000) than for those without (1.2 additional admissions per 100,000), despite a lower RR in people with type 2 diabetes.

Future research should focus on cause-specific hospital admissions to provide a more detailed comparison between people with and without diabetes. Such analyses could also consider the high burden of comorbidities in people with diabetes and assess how conditions such as, e.g. chronic kidney disease influence vulnerability to temperature extremes. Addressing these aspects may improve the prediction of heat and cold-related risks and support the development of more targeted adaptation measures for specific populations and diabetes subgroups. Moreover, future studies should include absolute effect measures to provide a more complete picture on the potential effect modification by different diabetes types as well as consider regional differences in temperature effects.

The main strength of our work is the large sample size which included the whole population in Germany. This enabled detailed analysis by several subgroups, in particular with regard to diabetes type. Moreover, this is the first study that compared the associations of air temperature on all-cause hospital admissions between people with and without diabetes. Further strengths include the long observation period covering 18 years (2005–2022) and the use of a consistent, harmonized, and validated meteorological dataset (E-OBS), which ensures comparability with other European studies. However, several limitations of our study should be considered when interpreting our findings. Exposure was assessed at the district level, which likely introduced measurement error. This is especially relevant in cities, where temperatures can vary substantially due to phenomena such as urban heat islands. More precise assessments use participants’ residential addresses, but that approach assumes individuals were at or near home during periods of heat or cold exposure, which is itself a limitation. Diabetes types were identified from claims data, which are documented for administrative rather than research purposes. This may have led to misclassification, as some individuals with diabetes might not have had their condition recorded as either primary or secondary diagnoses. Another limitation is that the data are available only at the case level, without individual identifiers. As a result, they cannot be linked to other sources, such as outpatient records, e.g. to validate diabetes status. The amount and restricted access to the data did not allow for extensive sensitivity analyses. For instance, the number and placement of knots in the splines of the DLNM usually should be varied to assess robustness of the results. Therefore, we relied on previous studies in this regard.^25^ We assume that these choices only have minor influence on the results, because previous studies which performed these sensitivity analyses generally show that the results are robust.^4^ In addition, the ecological design based on district-level counts precludes inference at the individual level, and ecological bias cannot be ruled out. Despite controlling for temporal and spatial trends, unmeasured and residual confounding by factors such as air pollution, humidity, infectious disease activity, or socioeconomic conditions may have influenced the results. On the other hand, these factors may also be partly determined by temperature and therefore rather act as mediators rather than confounders, such that adjusting for them could underestimate the total temperature effect. For instance, it has been argued that adjustment for air pollution may bias short-term effects of air temperature.^35^ Furthermore, temperature was linked to the day of admission rather than the onset of symptoms, which may have introduced temporal misalignment not fully captured by the distributed lag model. While the analysis focused on emergency admissions, temperature extremes might also affect the scheduling or postponement of elective procedures, indirectly influencing hospital admission patterns. Finally, the findings are specific to the German healthcare and climatic context and may not necessarily generalize to other regions or populations.

In summary, extreme heat was associated with an increased risk of all-cause emergency admission in almost all subgroups of age, sex and diabetes status. Extreme cold effects were protective in younger age groups but increased the risk in older age groups. Contrary to previous hypotheses, we found no evidence that people with diabetes are more vulnerable to temperature-related morbidity than people without diabetes.

## Contributors

TT conceptualized the study, performed the statistical analysis and wrote the initial draft of the manuscript. MW contributed to the statistical analysis. OK contributed to the statistical analysis and the conceptualization of the study. TT, MW, KPP, and OK contributed to the interpretation of data and to the critical revision of the manuscript for important intellectual content. Due to privacy protection rules, access to the DRG data was only possible indirectly and in close collaboration with the data holder. The coding and analyses were developed and tested by TT and MW using fully anonymized training datasets, which were equal in content and structure to the original data. These training datasets were accessed and verified by TT and MW. All authors had access to the training datasets, contributed to the interpretation of the results, and had final responsibility for the decision to submit the manuscript for publication.

## Data sharing statement

Environmental data are publicly available from the sources referenced in the manuscript. DRG data are available upon formal application from the institutions referenced in the manuscript.

## Editor note

The Lancet Group takes a neutral position with respect to territorial claims in published maps and institutional affiliations.

## Declaration of generative AI and AI-assisted technologies in the manuscript preparation process

During the preparation of this work the authors used ChatGPT (OpenAI) in order to revise parts of the text for readability. After using this tool/service, the authors reviewed and edited the content as needed and take full responsibility for the content of the published article.

## Declaration of interests

All authors declare no competing interests.
