# Supplementary material for: Air temperature and all-cause emergency hospital admissions in people with and without diabetes in Germany (2005–2022): a time-series analysis
Source: Lancet Reg Health Eur. 2026 Jan 23;63:101591. doi: 10.1016/j.lanepe.2026.101591 (PMC12951227; doi:10.1016/j.lanepe.2026.101591)
Supplement: Supplementary Figures [file mmc1.pdf]

Supplementary material

Air temperature and morbidity in people with and without Diabetes: A time-series analysis of 132 million emergency hospital admissions in Germany, 2005–2022

Thaddäus Tönnies Dr. PH[1]; Marielle Wirth MSc[1]; Katharina Piedboeuf-Potyka MSc[1]; Prof. Oliver Kuss [1,2,3]

[1] Institute for Biometrics and Epidemiology, German Diabetes Center, Leibniz Center for Diabetes Research at the Heinrich Heine University Düsseldorf, Düsseldorf, Nordrhein-Westfalen, Germany

[2] Centre for Health and Society, Medical Faculty and University Hospital Düsseldorf, Heinrich Heine University Düsseldorf, Düsseldorf, Germany

[3] German Center for Diabetes Research, München-Neuherberg, Germany

Table of contents

Figure S1. Lag-response association for extreme heat and all-cause emergency hospital admission by age, sex and diabetes status. 4

Figure S2. Lag-response association for extreme cold and all-cause emergency hospital admission by age, sex and diabetes status. 4

Figure S3. Overall cumulative association between daily air temperature and all-cause emergency hospital admission by age, sex and diabetes status – analysis restricted to pre-pandemic period, 2005-2019. 5

Figure S4. Overall cumulative association between extreme heat and all-cause emergency hospital admission by age, sex and diabetes status – analysis restricted to pre-pandemic period, 2005-2019. 6

Figure S5. Overall cumulative association between extreme cold and all-cause emergency hospital admission by age, sex and diabetes status – analysis restricted to pre-pandemic period, 2005-2019. 7

Figure S6. Overall cumulative association between daily air temperature and all-cause emergency hospital admission by age, sex and diabetes status – analysis restricted to rural areas. 8

Figure S7. Overall cumulative association between extreme heat and all-cause emergency hospital admission by age, sex and diabetes status– analysis restricted to rural areas. 9

Figure S8. Overall cumulative association between extreme cold and all-cause emergency hospital admission by age, sex and diabetes status – analysis restricted to rural areas. 10

Figure S9. Overall cumulative association between daily air temperature and all-cause emergency hospital admission by age, sex and diabetes status – analysis restricted to rural-urban areas. 11

Figure S10. Overall cumulative association between extreme heat and all-cause emergency hospital admission by age, sex and diabetes status – analysis restricted to rural-urban areas. 12

Figure S11. Overall cumulative association between extreme cold and all-cause emergency hospital admission by age, sex and diabetes status – analysis restricted to rural-urban areas. 13

Figure S12. Overall cumulative association between daily air temperature and all-cause emergency hospital admission by age, sex and diabetes status – analysis restricted to urban areas. 14

Figure S13. Overall cumulative association between extreme heat and all-cause emergency hospital admission by age, sex and diabetes status – analysis restricted to urban areas. 15

Figure S14. Overall cumulative association between extreme cold and all-cause emergency hospital admission by age, sex and diabetes status – analysis restricted to urban areas. 16

Figure S15. Overall cumulative association between daily air temperature and all-cause emergency hospital admission by age, sex and diabetes status – analysis modelling ten instead of 21 lag days. 17

Figure S16. Overall cumulative association between extreme heat and all-cause emergency hospital admission by age, sex and diabetes status – analysis modelling ten instead of 21 lag days. 18

Figure S17. Overall cumulative association between extreme cold and all-cause emergency hospital admission by age, sex and diabetes status – analysis modelling ten instead of 21 lag days. 19

Figure S18. Overall cumulative association between daily air temperature and all-cause emergency hospital admission by age, sex and diabetes status – analysis restricted to non-external causes for hospital admission. 20

Figure S19. Overall cumulative association between extreme heat and all-cause emergency hospital admission by age, sex and diabetes status – analysis restricted to non-external causes for hospital admission. 21

Figure S20. Overall cumulative association between extreme cold and all-cause emergency hospital admission by age, sex and diabetes status – analysis restricted to non-external causes for hospital admission. 22

Figure S21. Overall cumulative association between daily air temperature and all-cause emergency hospital admission by age, sex and diabetes status – analysis restricted to cause-specific diabetes hospital admissions. 23

Figure S22. Overall cumulative association between extreme heat and all-cause emergency hospital admission by age, sex and diabetes status – analysis restricted to cause-specific diabetes hospital admissions. 24

Figure S23. Overall cumulative association between extreme cold and all-cause emergency hospital admission by age, sex and diabetes status – analysis restricted to cause-specific diabetes hospital admissions. 25

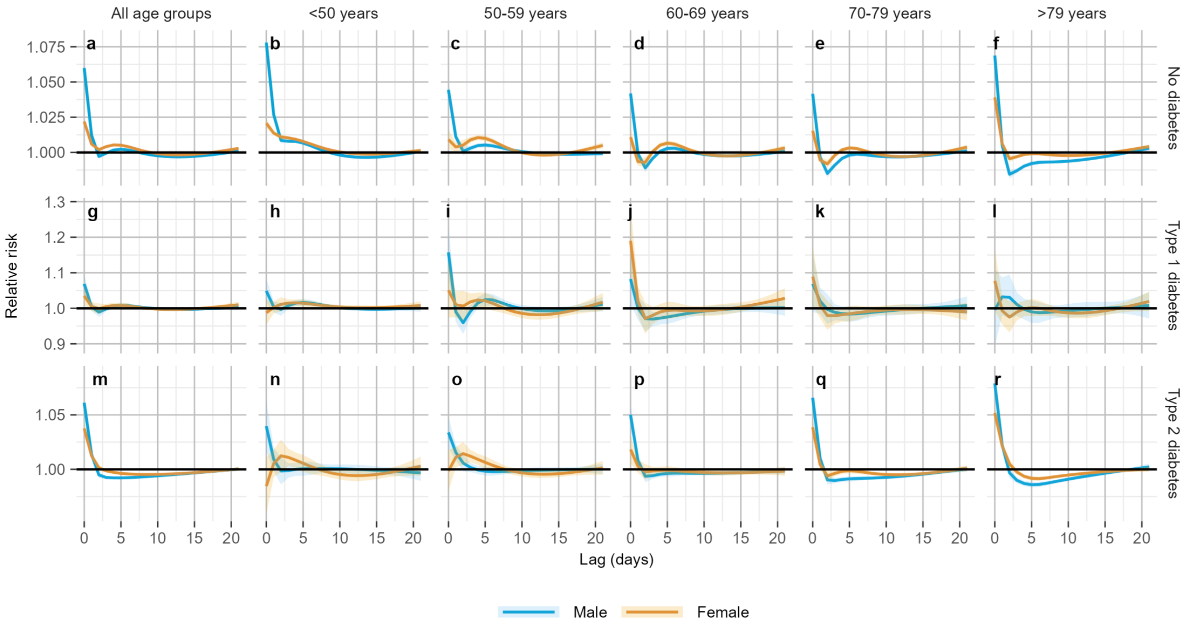

Figure S1. Lag-response association for extreme heat and all-cause emergency hospital admission by age, sex and diabetes status. Extreme heat was defined as daily average temperature of 24.5 °C (99th percentile of the daily temperature distribution). The panels show results for people without diabetes (a-f), with type 1 diabetes (g-l), type 2 diabetes (m-r) including all age groups (a, g, m), age < 50 years (b, h, n), 50-59 years (c, i, o), 60-69 years (d, j, p), 70-79 years (e, k, q) and > 79 years (f, l, r). Estimates are based on separate conditional quasi-Poisson regressions with distributed lag non-linear models including lag days 0 to 21.

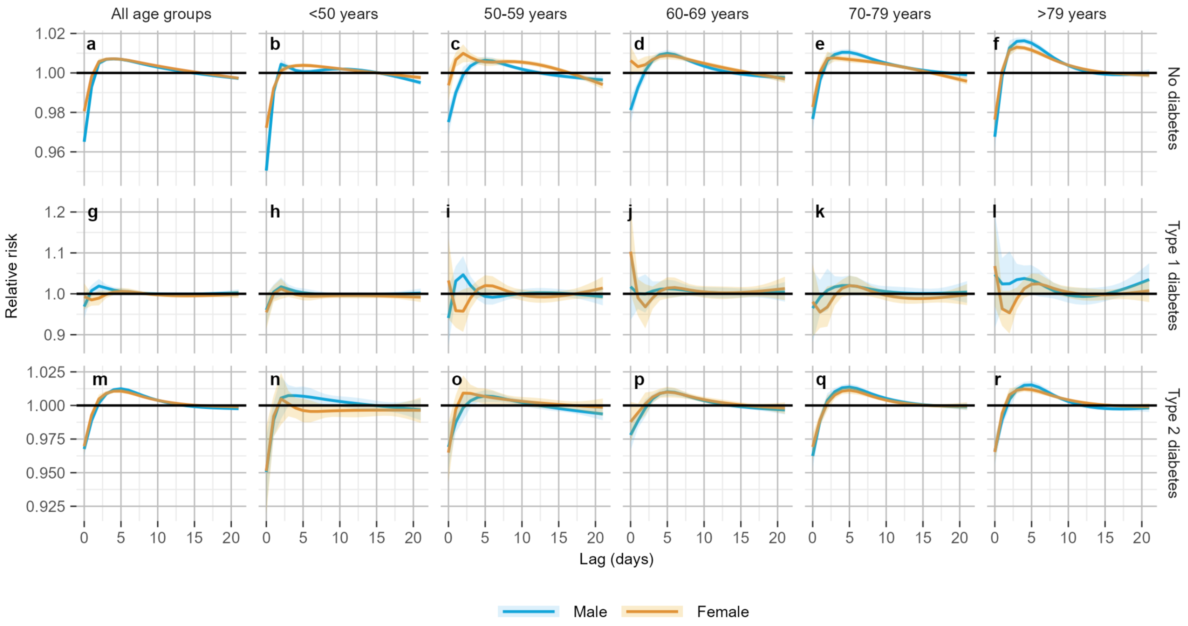

Figure S2. Lag-response association for extreme cold and all-cause emergency hospital admission by age, sex and diabetes status. Extreme cold was defined as daily average temperature of -6.5 °C (1st percentile of the daily temperature distribution). The panels show results for people without diabetes (a-f), with type 1 diabetes (g-l), type 2 diabetes (m-r) including all age groups (a, g, m), age < 50 years (b, h, n), 50-59 years (c, i, o), 60-69 years (d, j, p), 70-79 years (e, k, q) and > 79 years (f, l, r). Estimates are based on separate conditional quasi-Poisson regressions with distributed lag non-linear models including lag days 0 to 21.

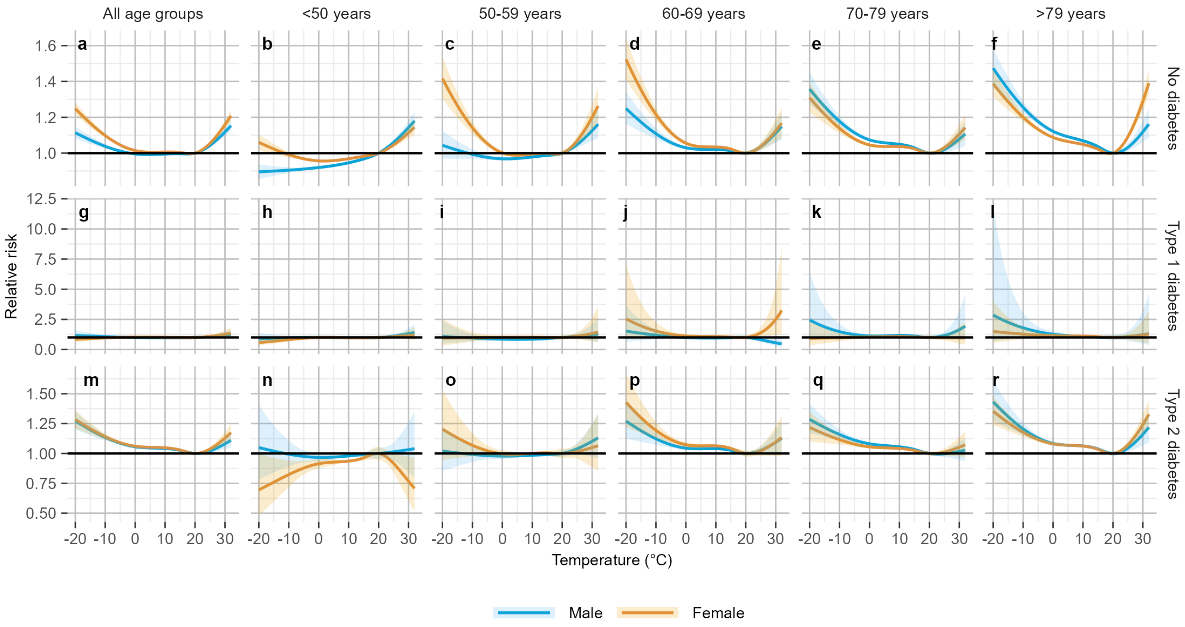

Figure S3. Overall cumulative association between daily air temperature and all-cause emergency hospital admission by age, sex and diabetes status – analysis restricted to pre-pandemic period, 2005-2019. The panels show results for people without diabetes (a-f), with type 1 diabetes (g-l), type 2 diabetes (m-r) including all age groups (a, g, m), age < 50 years (b, h, n), 50-59 years (c, i, o), 60-69 years (d, j, p), 70-79 years (e, k, q) and > 79 years (f, l, r). Estimates are based on separate conditional quasi-Poisson regressions with distributed lag non-linear models including lag days 0 to 21.

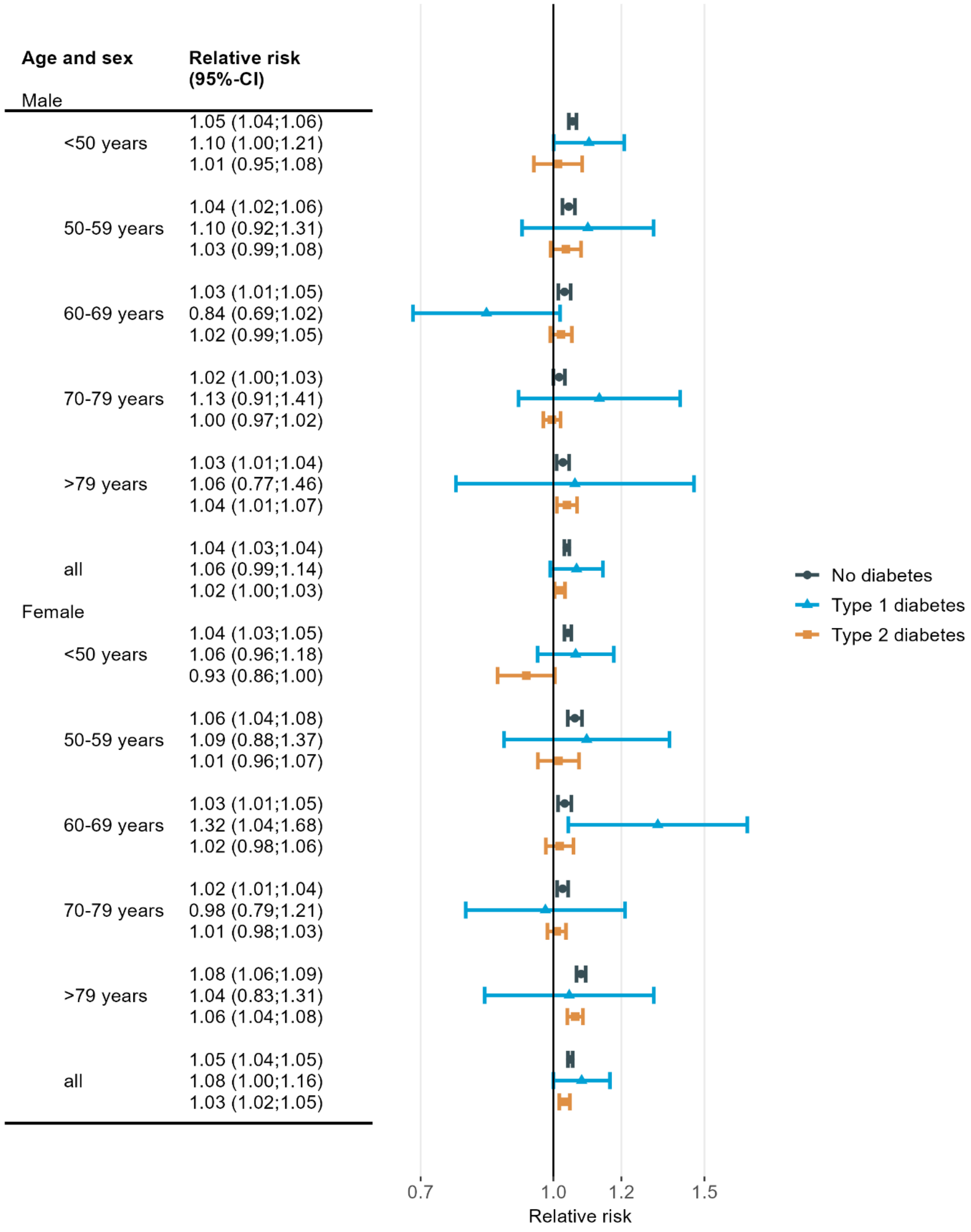

Figure S4. Overall cumulative association between extreme heat and all-cause emergency hospital admission by age, sex and diabetes status – analysis restricted to pre-pandemic period, 2005-2019.

Age and sex	Relative risk (95%-CI)
Male	
<50 years	0.91 (0.90;0.92)
	0.99 (0.90;1.08)
	0.98 (0.92;1.05)
50-59 years	0.98 (0.96;1.00)
	0.90 (0.75;1.08)
	0.98 (0.95;1.02)
60-69 years	1.07 (1.06;1.09)
	1.11 (0.91;1.35)
	1.09 (1.06;1.12)
70-79 years	1.13 (1.11;1.15)
	1.29 (1.05;1.60)
	1.12 (1.10;1.15)
>79 years	1.20 (1.18;1.22)
	1.56 (1.13;2.15)
	1.15 (1.12;1.18)
all	1.02 (1.01;1.02)
	1.04 (0.97;1.11)
	1.10 (1.08;1.11)
Female	
<50 years	0.97 (0.97;0.98)
	0.90 (0.81;1.00)
	0.86 (0.80;0.94)
50-59 years	1.08 (1.06;1.10)
	1.00 (0.79;1.25)
	1.03 (0.98;1.09)
60-69 years	1.14 (1.12;1.16)
	1.33 (1.05;1.68)
	1.14 (1.10;1.18)
70-79 years	1.09 (1.08;1.11)
	1.01 (0.82;1.24)
	1.09 (1.06;1.11)
>79 years	1.15 (1.14;1.16)
	1.22 (0.97;1.53)
	1.13 (1.11;1.15)
all	1.06 (1.05;1.07)
	0.99 (0.92;1.07)
	1.10 (1.09;1.12)

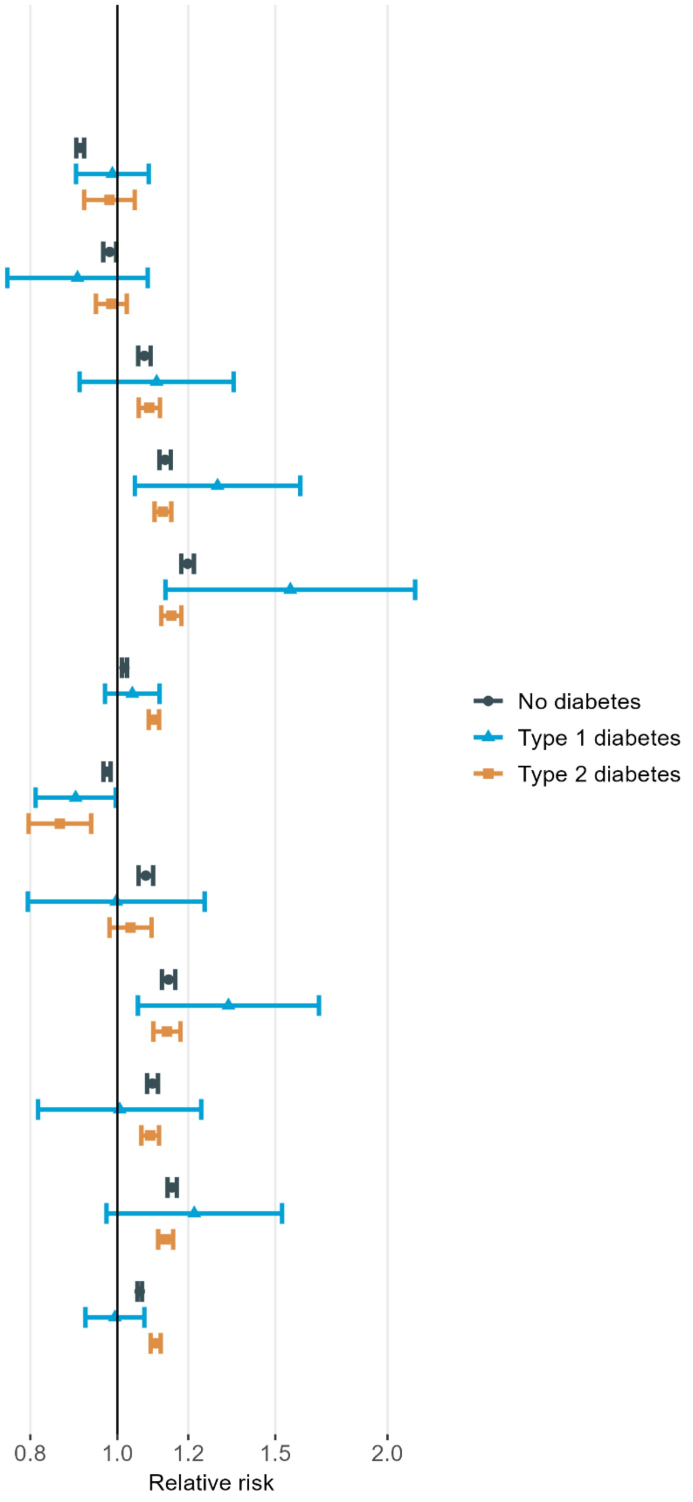

Figure S5. Overall cumulative association between extreme cold and all-cause emergency hospital admission by age, sex and diabetes status – analysis restricted to pre-pandemic period, 2005-2019.

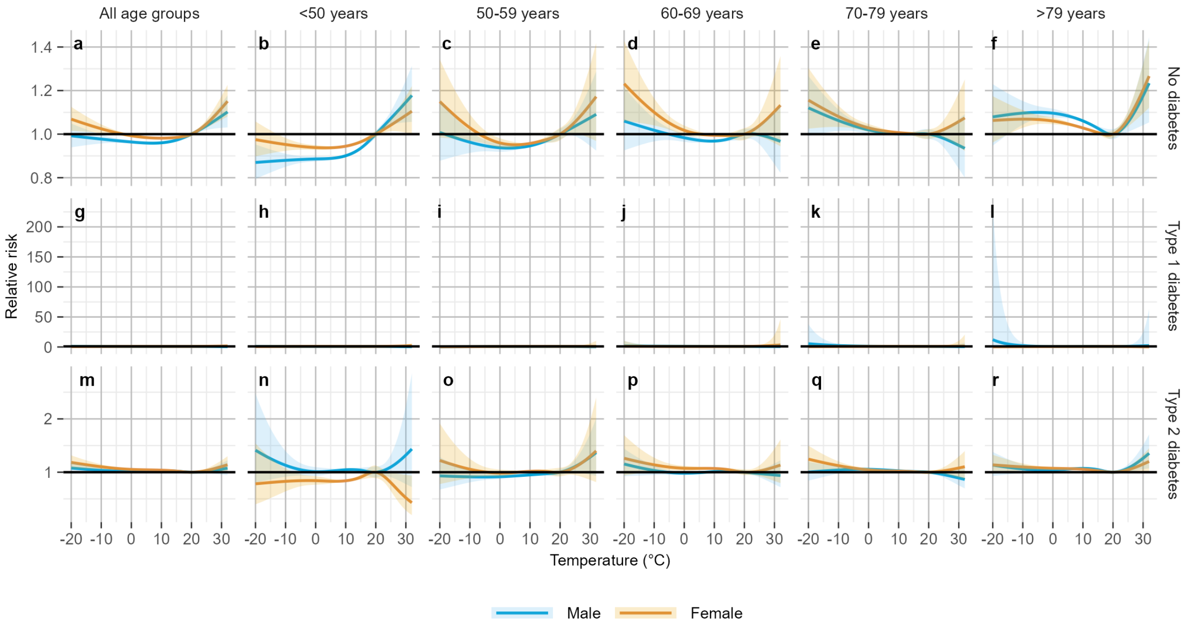

Figure S6. Overall cumulative association between daily air temperature and all-cause emergency hospital admission by age, sex and diabetes status – analysis restricted to rural areas. The panels show results for people without diabetes (a-f), with type 1 diabetes (g-l), type 2 diabetes (m-r) including all age groups (a, g, m), age < 50 years (b, h, n), 50-59 years (c, i, o), 60-69 years (d, j, p), 70-79 years (e, k, q) and > 79 years (f, l, r). Estimates are based on separate conditional quasi-Poisson regressions with distributed lag non-linear models including lag days 0 to 21.

Age and sex	Relative risk (95%-CI)
Male	
	1.06 (1.04;1.09)
<50 years	0.86 (0.68;1.09)
	1.08 (0.92;1.28)
	1.03 (0.99;1.07)
50-59 years	0.79 (0.52;1.20)
	1.09 (1.00;1.20)
	1.00 (0.96;1.04)
60-69 years	0.92 (0.56;1.51)
	0.98 (0.92;1.04)
	0.98 (0.95;1.02)
70-79 years	0.85 (0.46;1.56)
	0.96 (0.91;1.01)
	1.05 (1.01;1.09)
>79 years	1.19 (0.53;2.68)
	1.06 (1.01;1.12)
	1.03 (1.02;1.05)
all	0.87 (0.73;1.04)
	1.01 (0.98;1.04)
Female	
	1.04 (1.01;1.06)
<50 years	1.23 (0.95;1.60)
	0.83 (0.69;1.01)
	1.05 (1.00;1.10)
50-59 years	1.02 (0.59;1.75)
	1.08 (0.95;1.23)
	1.03 (0.99;1.08)
60-69 years	1.40 (0.73;2.68)
	1.02 (0.93;1.11)
	1.02 (0.98;1.06)
70-79 years	1.12 (0.61;2.05)
	1.02 (0.97;1.08)
	1.06 (1.03;1.09)
>79 years	0.74 (0.38;1.44)
	1.04 (0.99;1.09)
	1.04 (1.03;1.06)
all	1.16 (0.95;1.41)
	1.03 (0.99;1.06)

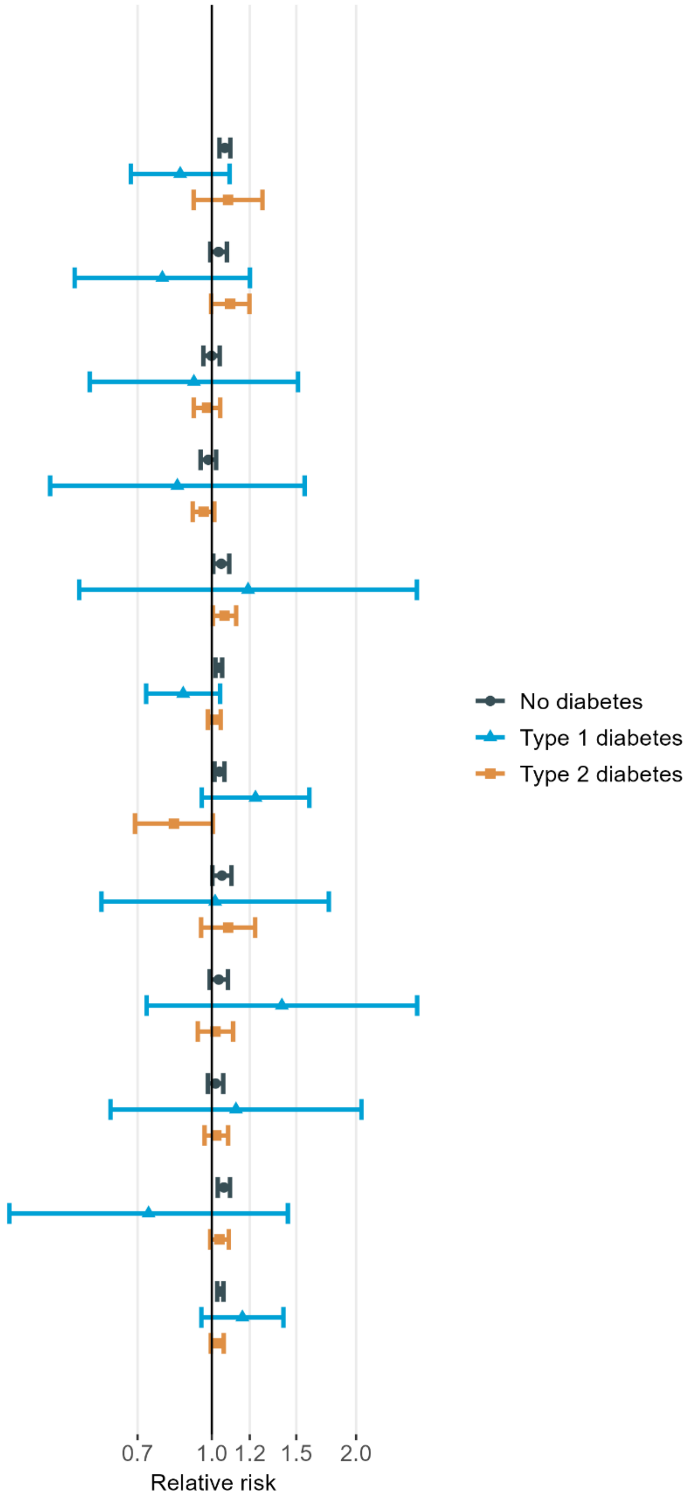

Figure S7. Overall cumulative association between extreme heat and all-cause emergency hospital admission by age, sex and diabetes status– analysis restricted to rural areas.

Age and sex		Relative risk (95%-CI)
Male		
	<50 years	0.88 (0.86;0.90)
		0.90 (0.73;1.10)
		1.07 (0.93;1.22)
	50-59 years	0.95 (0.92;0.98)
		0.61 (0.42;0.88)
		0.91 (0.84;0.98)
	60-69 years	1.00 (0.97;1.04)
		1.41 (0.91;2.18)
		1.00 (0.95;1.06)
	70-79 years	1.04 (1.02;1.08)
		1.88 (1.15;3.06)
		1.05 (1.01;1.09)
	>79 years	1.10 (1.07;1.13)
		1.36 (0.66;2.81)
		1.04 (1.00;1.09)
	all	0.97 (0.96;0.99)
		0.97 (0.84;1.13)
		1.02 (1.00;1.05)
Female		
	<50 years	0.95 (0.93;0.96)
		1.00 (0.80;1.24)
		0.84 (0.71;0.98)
	50-59 years	1.00 (0.96;1.03)
		0.70 (0.44;1.11)
		1.02 (0.92;1.13)
	60-69 years	1.06 (1.03;1.10)
		0.95 (0.57;1.57)
		1.11 (1.03;1.19)
	70-79 years	1.06 (1.03;1.09)
		0.85 (0.52;1.39)
		1.09 (1.04;1.14)
	>79 years	1.07 (1.04;1.09)
		0.58 (0.32;1.03)
		1.09 (1.05;1.13)
	all	1.01 (1.00;1.02)
		0.90 (0.76;1.06)
		1.08 (1.05;1.11)

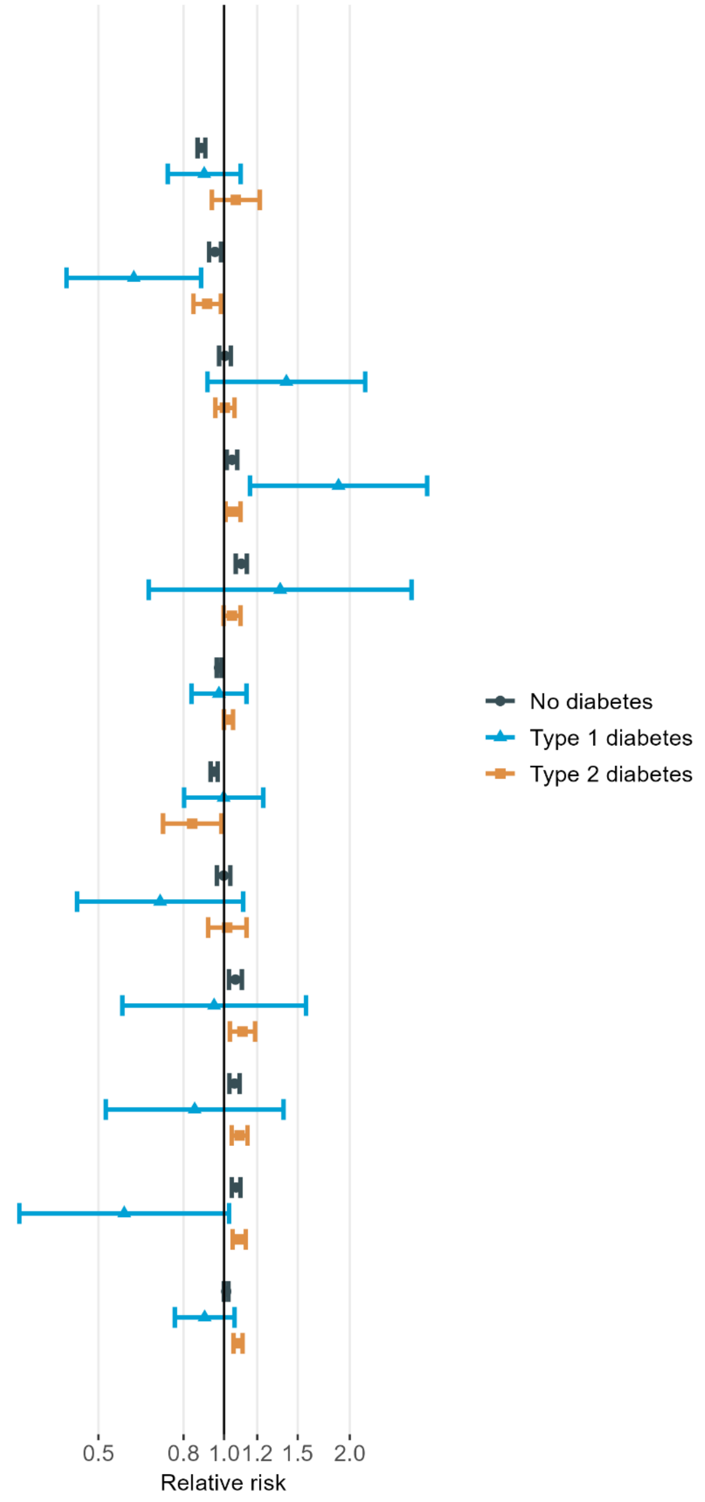

Figure S8. Overall cumulative association between extreme cold and all-cause emergency hospital admission by age, sex and diabetes status – analysis restricted to rural areas.

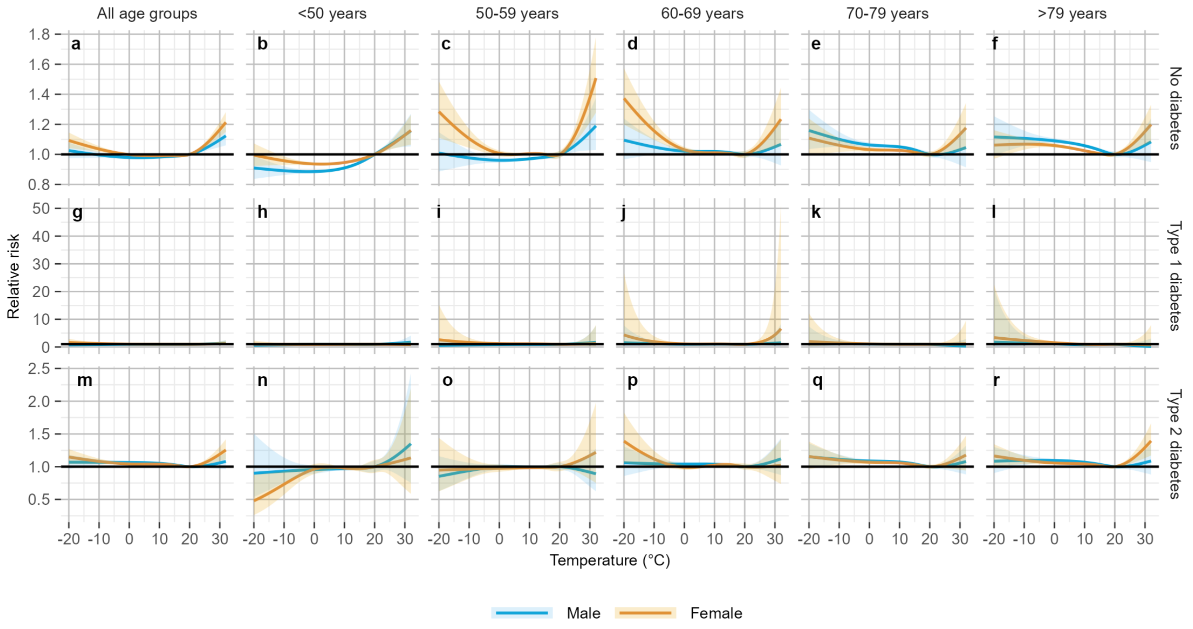

Figure S9. Overall cumulative association between daily air temperature and all-cause emergency hospital admission by age, sex and diabetes status – analysis restricted to rural-urban areas. The panels show results for people without diabetes (a-f), with type 1 diabetes (g-l), type 2 diabetes (m-r) including all age groups (a, g, m), age < 50 years (b, h, n), 50-59 years (c, i, o), 60-69 years (d, j, p), 70-79 years (e, k, q) and > 79 years (f, l, r). Estimates are based on separate conditional quasi-Poisson regressions with distributed lag non-linear models including lag days 0 to 21.

Age and sex	Relative risk (95%-CI)
Male	
	1.06 (1.03;1.08)
<50 years	1.16 (0.94;1.43)
	1.09 (0.94;1.25)
	1.05 (1.01;1.09)
50-59 years	1.16 (0.80;1.67)
	0.97 (0.89;1.06)
	1.01 (0.98;1.05)
60-69 years	1.10 (0.72;1.66)
	1.02 (0.96;1.08)
	1.00 (0.97;1.03)
70-79 years	0.75 (0.44;1.25)
	1.01 (0.96;1.06)
	1.01 (0.98;1.04)
>79 years	0.69 (0.35;1.36)
	1.01 (0.96;1.06)
	1.03 (1.02;1.05)
all	1.08 (0.93;1.26)
	1.01 (0.98;1.04)
Female	
	1.05 (1.03;1.07)
<50 years	0.97 (0.78;1.21)
	1.04 (0.89;1.23)
	1.11 (1.06;1.15)
50-59 years	1.02 (0.65;1.59)
	1.06 (0.94;1.19)
	1.05 (1.01;1.09)
60-69 years	1.58 (0.95;2.63)
	0.99 (0.92;1.08)
	1.04 (1.00;1.07)
70-79 years	1.04 (0.62;1.75)
	1.03 (0.98;1.09)
	1.05 (1.02;1.07)
>79 years	0.99 (0.57;1.72)
	1.08 (1.03;1.13)
	1.05 (1.04;1.07)
all	1.04 (0.89;1.23)
	1.05 (1.02;1.08)

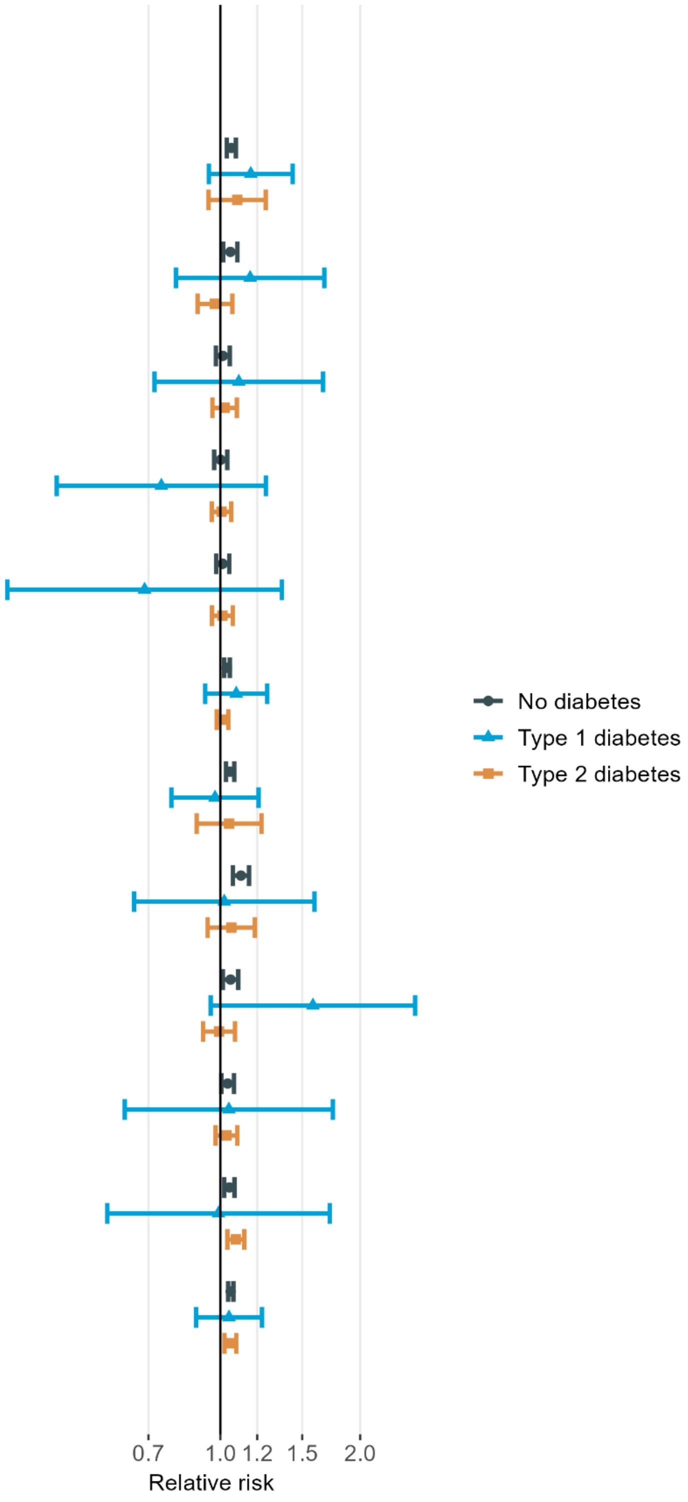

Figure S10. Overall cumulative association between extreme heat and all-cause emergency hospital admission by age, sex and diabetes status – analysis restricted to rural-urban areas.

Age and sex	Relative risk (95%-CI)
**Male**	
<50 years	0.89 (0.87;0.90)
	0.91 (0.76;1.09)
	0.94 (0.83;1.07)
50-59 years	0.97 (0.94;1.00)
	0.83 (0.59;1.15)
	0.96 (0.90;1.04)
60-69 years	1.04 (1.01;1.07)
	1.09 (0.74;1.62)
	1.04 (0.99;1.10)
70-79 years	1.08 (1.05;1.11)
	1.12 (0.73;1.74)
	1.10 (1.06;1.15)
>79 years	1.10 (1.07;1.13)
	1.34 (0.71;2.54)
	1.10 (1.05;1.15)
all	0.99 (0.98;1.00)
	0.95 (0.83;1.09)
	1.07 (1.04;1.09)
**Female**	
<50 years	0.95 (0.93;0.96)
	0.96 (0.79;1.16)
	0.83 (0.71;0.96)
50-59 years	1.06 (1.02;1.10)
	1.44 (0.94;2.21)
	0.97 (0.87;1.07)
60-69 years	1.10 (1.06;1.14)
	1.51 (0.96;2.35)
	1.05 (0.99;1.13)
70-79 years	1.05 (1.02;1.08)
	1.31 (0.84;2.05)
	1.09 (1.04;1.13)
>79 years	1.07 (1.04;1.09)
	2.06 (1.28;3.31)
	1.08 (1.04;1.12)
all	1.02 (1.01;1.03)
	1.17 (1.01;1.35)
	1.06 (1.04;1.09)

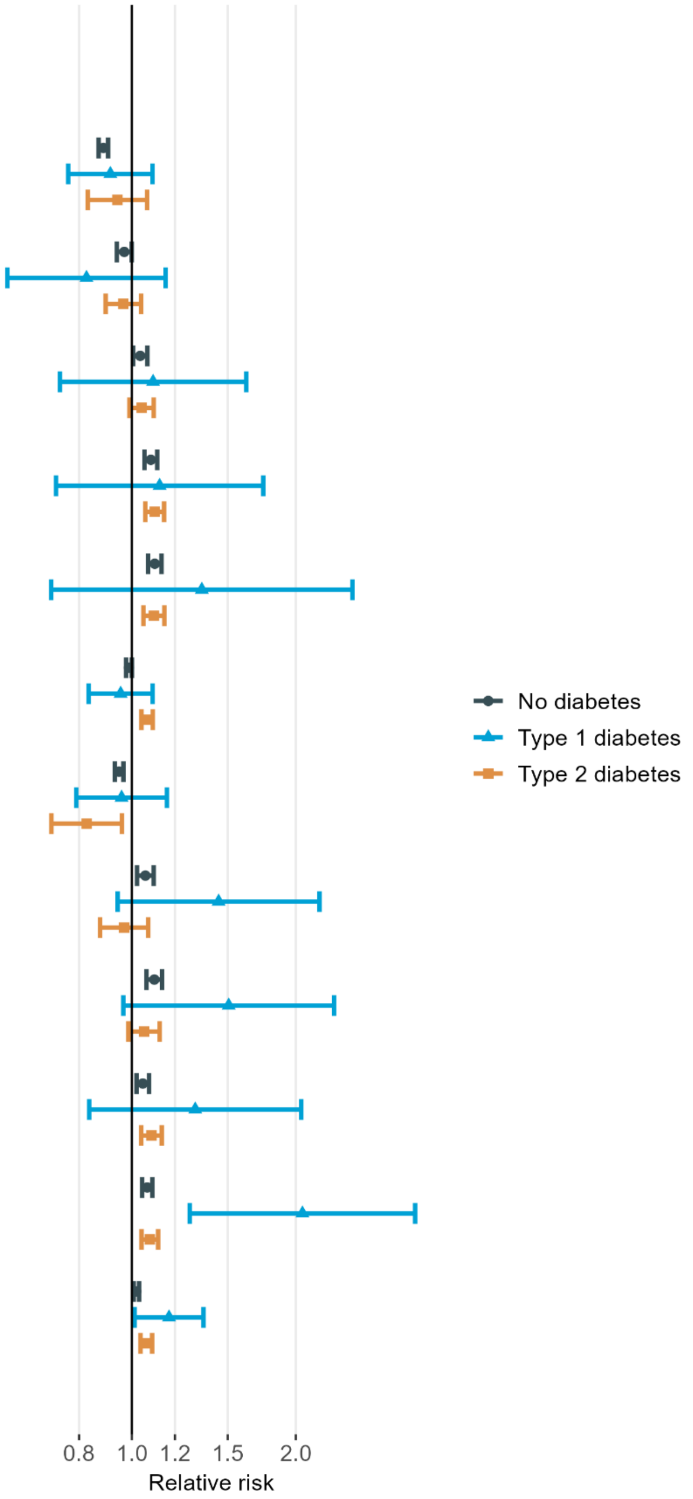

Figure S11. Overall cumulative association between extreme cold and all-cause emergency hospital admission by age, sex and diabetes status – analysis restricted to rural-urban areas.

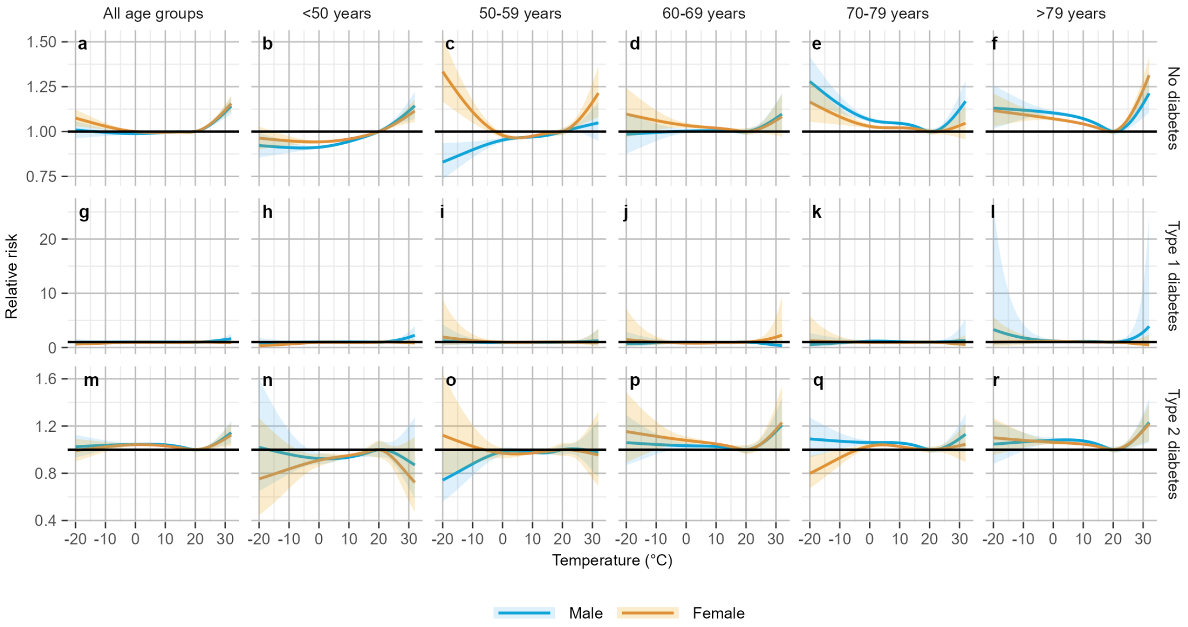

Figure S12. Overall cumulative association between daily air temperature and all-cause emergency hospital admission by age, sex and diabetes status – analysis restricted to urban areas. The panels show results for people without diabetes (a-f), with type 1 diabetes (g-l), type 2 diabetes (m-r) including all age groups (a, g, m), age < 50 years (b, h, n), 50-59 years (c, i, o), 60-69 years (d, j, p), 70-79 years (e, k, q) and > 79 years (f, l, r). Estimates are based on separate conditional quasi-Poisson regressions with distributed lag non-linear models including lag days 0 to 21.

Age and sex	Relative risk (95%-CI)
Male	
	1.04 (1.03;1.06)
<50 years	1.21 (1.07;1.37)
	0.98 (0.90;1.07)
	1.02 (1.00;1.04)
50-59 years	1.07 (0.84;1.35)
	1.01 (0.95;1.06)
	1.02 (1.00;1.04)
60-69 years	0.81 (0.62;1.07)
	1.04 (1.00;1.08)
	1.03 (1.00;1.05)
70-79 years	1.07 (0.78;1.46)
	1.02 (0.98;1.05)
	1.03 (1.01;1.05)
>79 years	1.32 (0.87;2.01)
	1.03 (1.00;1.07)
	1.03 (1.02;1.04)
all	1.12 (1.02;1.24)
	1.02 (1.00;1.04)
Female	
	1.03 (1.02;1.05)
<50 years	1.01 (0.89;1.15)
	0.93 (0.85;1.03)
	1.05 (1.02;1.08)
50-59 years	0.98 (0.74;1.32)
	0.99 (0.92;1.07)
	1.02 (0.99;1.04)
60-69 years	1.25 (0.90;1.73)
	1.04 (0.99;1.09)
	1.00 (0.98;1.02)
70-79 years	0.89 (0.65;1.21)
	1.01 (0.97;1.04)
	1.06 (1.04;1.08)
>79 years	0.86 (0.61;1.21)
	1.04 (1.01;1.07)
	1.04 (1.03;1.04)
all	1.00 (0.91;1.11)
	1.02 (1.00;1.04)

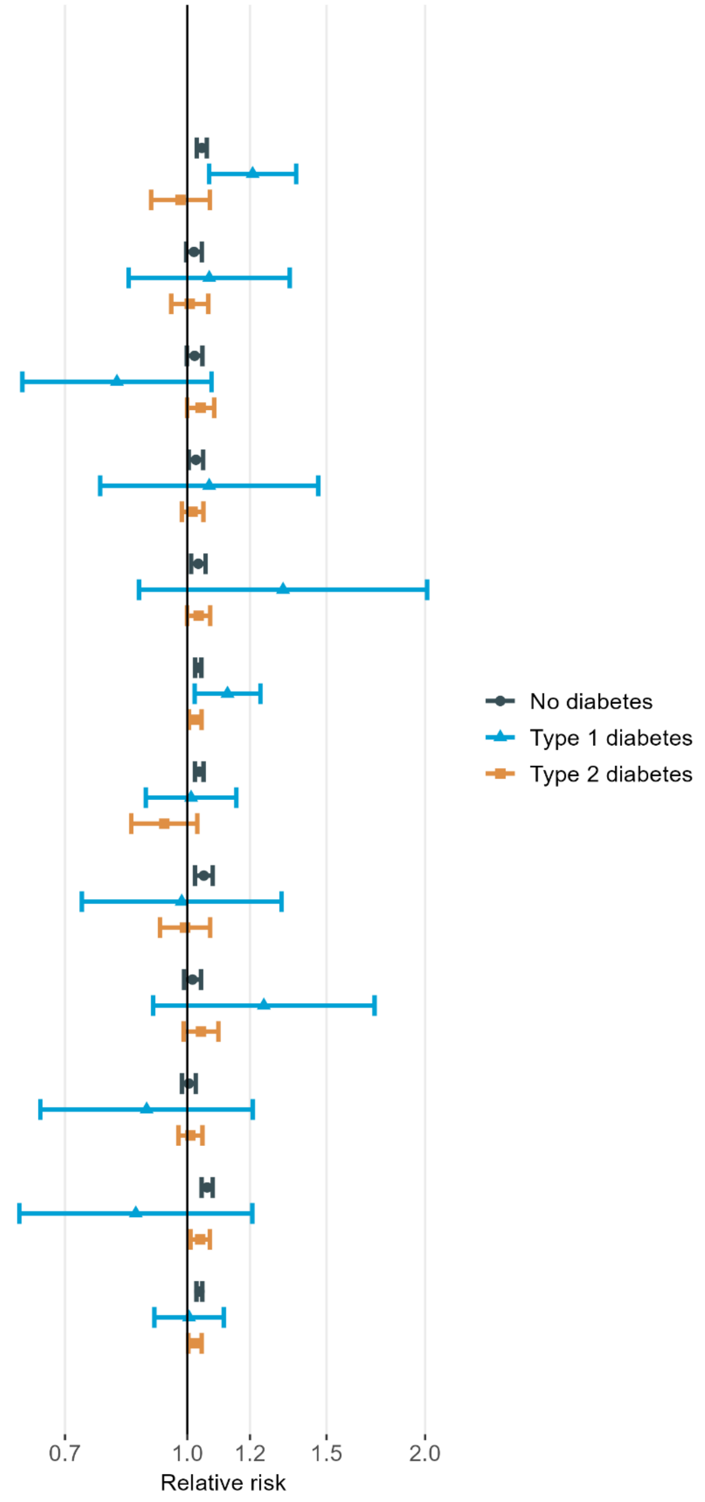

Figure S13. Overall cumulative association between extreme heat and all-cause emergency hospital admission by age, sex and diabetes status – analysis restricted to urban areas.

Age and sex	Relative risk (95%-CI)
Male	
	0.91 (0.89;0.93)
<50 years	0.98 (0.82;1.16)
	0.94 (0.84;1.06)
	0.92 (0.89;0.95)
50-59 years	0.96 (0.69;1.33)
	0.93 (0.86;1.00)
	1.00 (0.97;1.03)
60-69 years	0.91 (0.63;1.33)
	1.04 (0.99;1.09)
	1.11 (1.08;1.14)
70-79 years	1.00 (0.66;1.52)
	1.07 (1.02;1.11)
	1.11 (1.08;1.15)
>79 years	1.48 (0.86;2.55)
	1.07 (1.02;1.12)
	0.99 (0.98;1.00)
all	0.99 (0.87;1.13)
	1.04 (1.02;1.07)
Female	
	0.94 (0.93;0.96)
<50 years	0.77 (0.64;0.92)
	0.87 (0.75;0.99)
	1.05 (1.01;1.09)
50-59 years	1.15 (0.77;1.71)
	1.00 (0.91;1.11)
	1.05 (1.02;1.09)
60-69 years	0.92 (0.60;1.42)
	1.10 (1.03;1.18)
	1.06 (1.03;1.09)
70-79 years	1.02 (0.68;1.54)
	0.97 (0.93;1.02)
	1.09 (1.06;1.11)
>79 years	1.15 (0.73;1.81)
	1.07 (1.03;1.11)
	1.02 (1.00;1.03)
all	0.88 (0.77;1.01)
	1.03 (1.01;1.06)

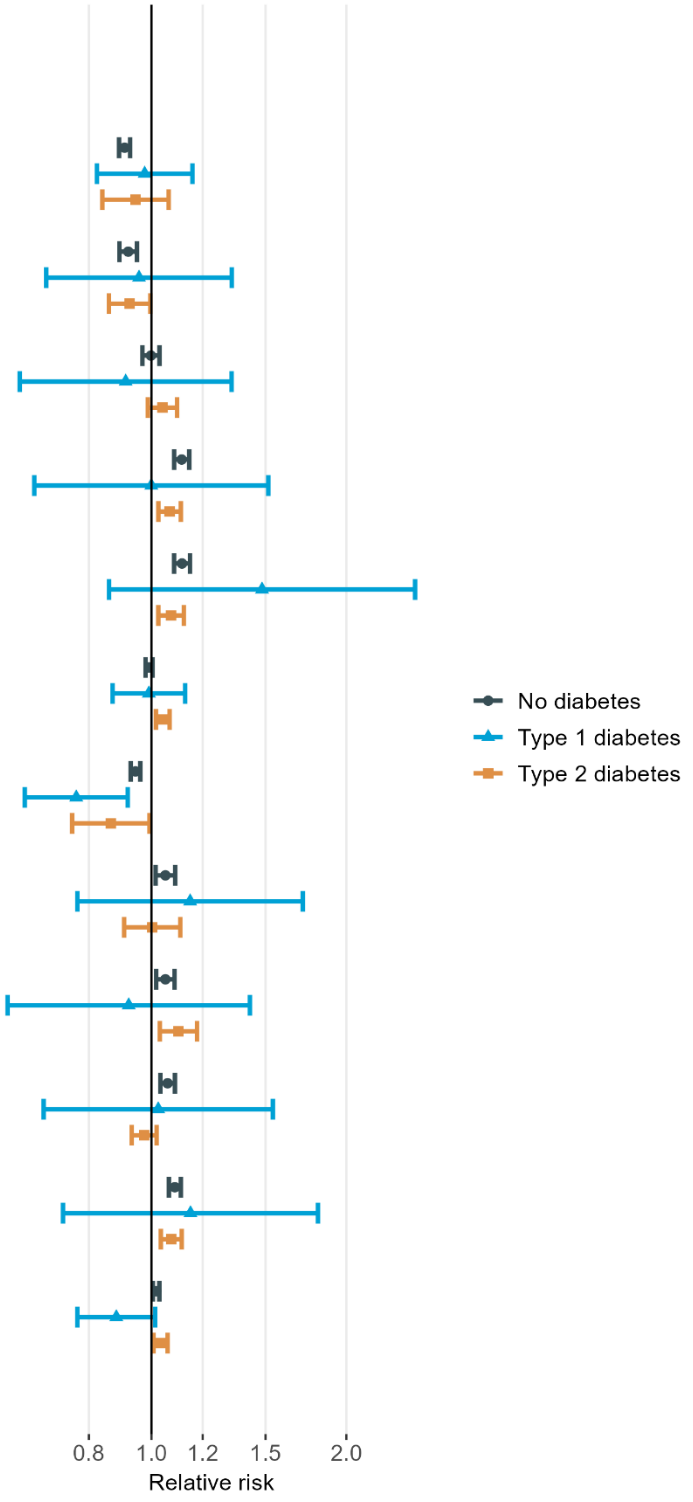

Figure S14. Overall cumulative association between extreme cold and all-cause emergency hospital admission by age, sex and diabetes status – analysis restricted to urban areas.

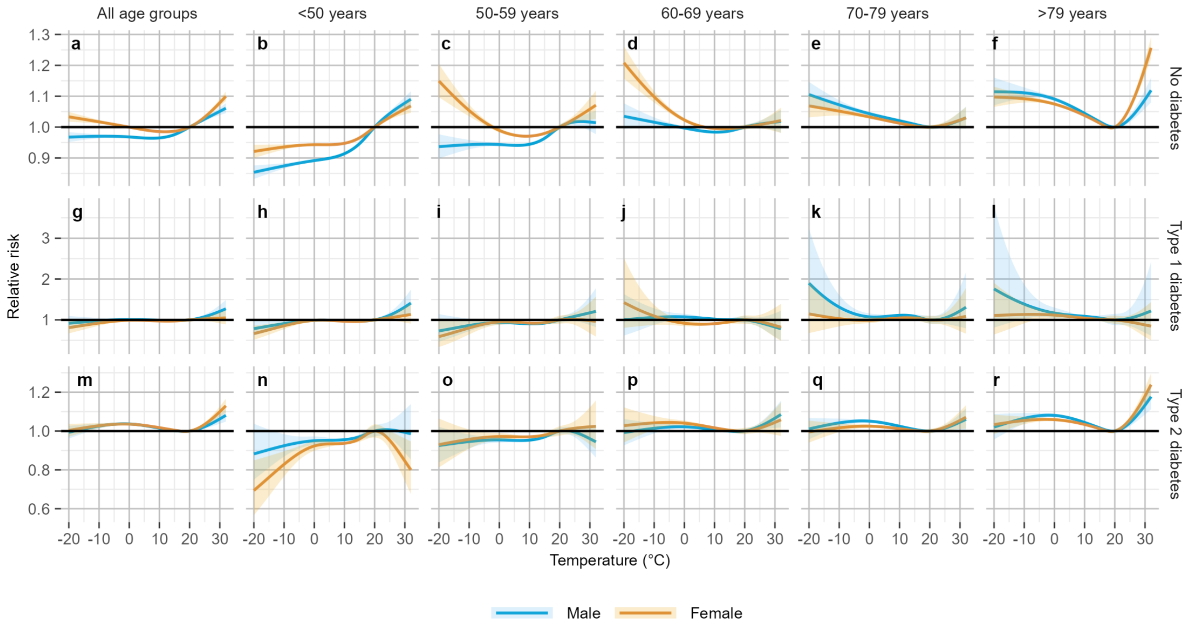

Figure S15. Overall cumulative association between daily air temperature and all-cause emergency hospital admission by age, sex and diabetes status – analysis modelling ten instead of 21 lag days. The panels show results for people without diabetes (a-f), with type 1 diabetes (g-l), type 2 diabetes (m-r) including all age groups (a, g, m), age < 50 years (b, h, n), 50-59 years (c, i, o), 60-69 years (d, j, p), 70-79 years (e, k, q) and > 79 years (f, l, r). Estimates are based on separate conditional quasi-Poisson regressions with distributed lag non-linear models including lag days 0 to 21.

Age and sex	Relative risk (95%-CI)
Male	
	1.04 (1.03;1.05)
<50 years	1.10 (1.04;1.16)
	1.01 (0.97;1.05)
	1.02 (1.01;1.03)
50-59 years	1.08 (0.97;1.20)
	1.00 (0.97;1.02)
	1.01 (1.00;1.02)
60-69 years	0.94 (0.84;1.06)
	1.02 (1.00;1.04)
	1.00 (1.00;1.01)
70-79 years	1.04 (0.91;1.18)
	1.01 (1.00;1.03)
	1.02 (1.01;1.03)
>79 years	1.03 (0.86;1.25)
	1.04 (1.02;1.05)
	1.02 (1.02;1.03)
all	1.07 (1.02;1.11)
	1.02 (1.01;1.03)
Female	
	1.03 (1.02;1.03)
<50 years	1.05 (0.99;1.11)
	0.96 (0.92;1.01)
	1.02 (1.01;1.04)
50-59 years	1.02 (0.89;1.16)
	1.01 (0.98;1.05)
	1.01 (1.00;1.02)
60-69 years	0.97 (0.84;1.12)
	1.01 (0.99;1.04)
	1.01 (1.00;1.02)
70-79 years	1.00 (0.88;1.14)
	1.02 (1.00;1.03)
	1.05 (1.05;1.06)
>79 years	0.96 (0.83;1.10)
	1.05 (1.04;1.06)
	1.03 (1.02;1.03)
all	1.02 (0.98;1.07)
	1.03 (1.02;1.04)

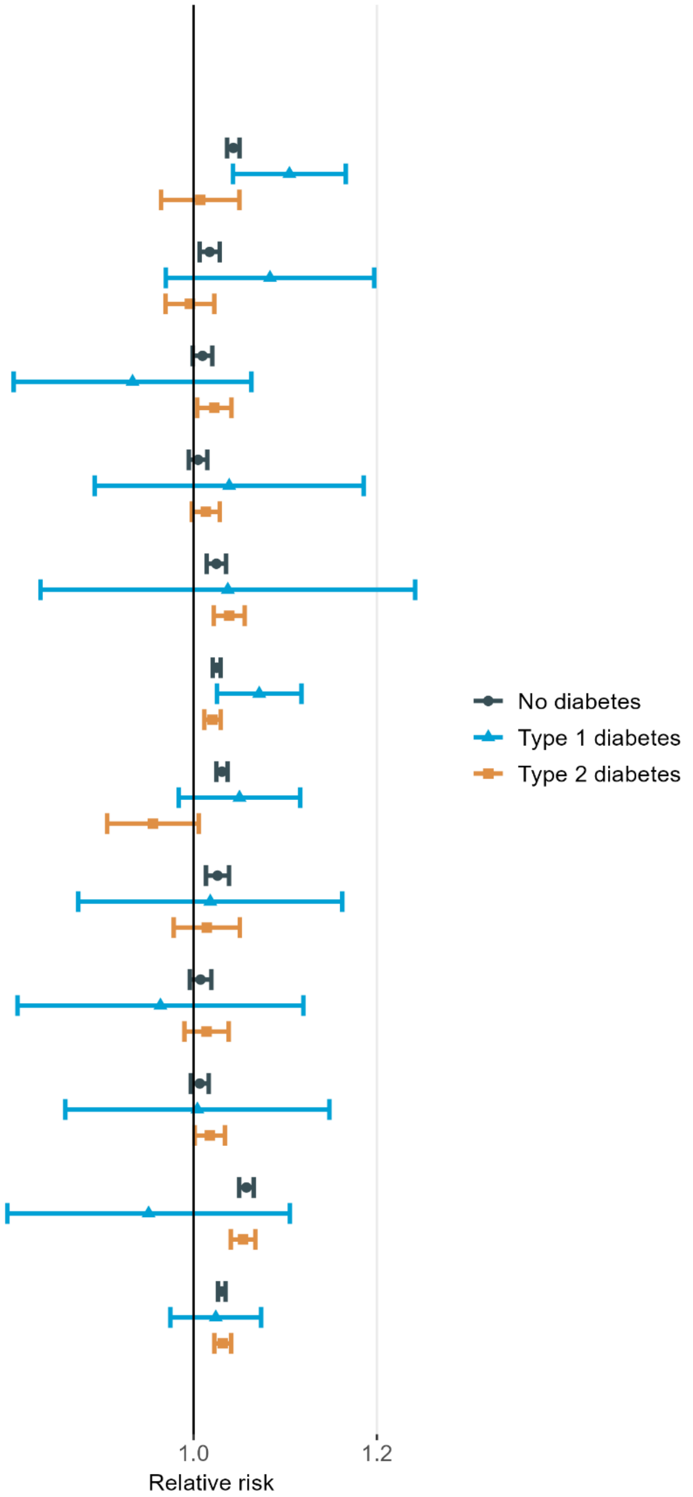

Figure S16. Overall cumulative association between extreme heat and all-cause emergency hospital admission by age, sex and diabetes status – analysis modelling ten instead of 21 lag days.

Age and sex	Relative risk (95%-CI)
**Male**	
<50 years	0.88 (0.88;0.89)
	0.95 (0.89;1.00)
	0.94 (0.90;0.97)
50-59 years	0.94 (0.93;0.95)
	0.90 (0.81;1.01)
	0.95 (0.93;0.97)
60-69 years	1.01 (1.00;1.02)
	1.07 (0.95;1.21)
	1.02 (1.00;1.04)
70-79 years	1.06 (1.05;1.07)
	1.19 (1.05;1.35)
	1.05 (1.04;1.06)
>79 years	1.11 (1.10;1.12)
	1.29 (1.07;1.56)
	1.08 (1.06;1.09)
all	0.97 (0.97;0.97)
	0.99 (0.95;1.04)
	1.03 (1.02;1.04)
**Female**	
<50 years	0.94 (0.93;0.94)
	0.92 (0.86;0.98)
	0.87 (0.83;0.91)
50-59 years	1.03 (1.02;1.04)
	0.87 (0.76;1.00)
	0.96 (0.93;1.00)
60-69 years	1.07 (1.06;1.08)
	1.01 (0.88;1.17)
	1.04 (1.02;1.07)
70-79 years	1.05 (1.04;1.05)
	1.01 (0.88;1.14)
	1.02 (1.01;1.04)
>79 years	1.09 (1.08;1.10)
	1.13 (0.99;1.30)
	1.06 (1.05;1.07)
all	1.01 (1.01;1.01)
	0.96 (0.91;1.00)
	1.03 (1.02;1.04)

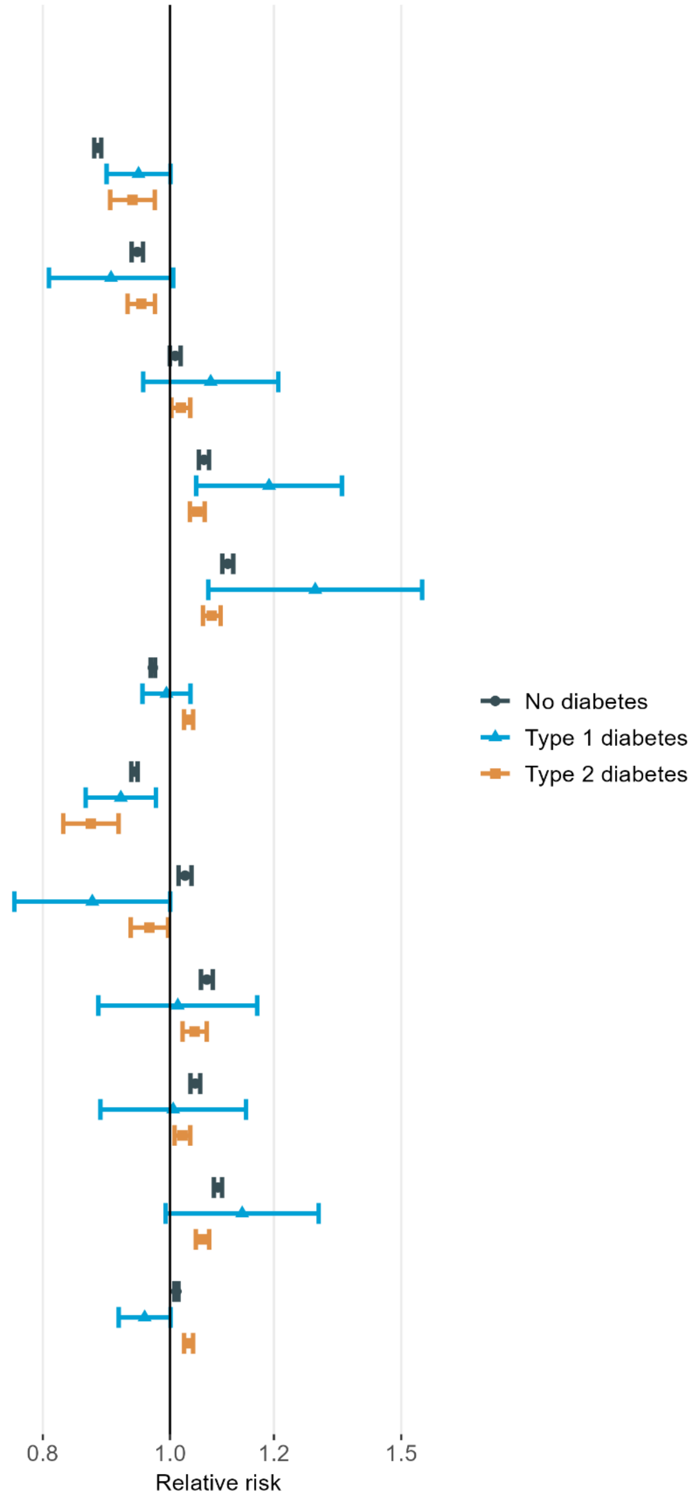

Figure S17. Overall cumulative association between extreme cold and all-cause emergency hospital admission by age, sex and diabetes status – analysis modelling ten instead of 21 lag days.

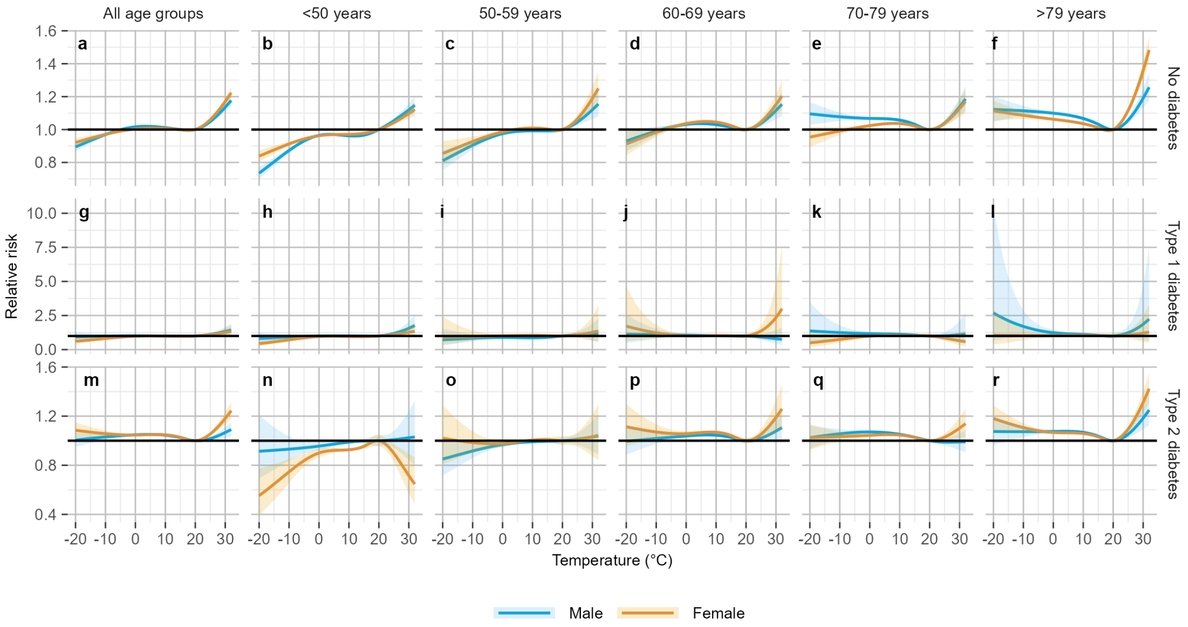

Figure S18. Overall cumulative association between daily air temperature and all-cause emergency hospital admission by age, sex and diabetes status – analysis restricted to non-external causes for hospital admission. The panels show results for people without diabetes (a-f), with type 1 diabetes (g-l), type 2 diabetes (m-r) including all age groups (a, g, m), age < 50 years (b, h, n), 50-59 years (c, i, o), 60-69 years (d, j, p), 70-79 years (e, k, q) and > 79 years (f, l, r). Estimates are based on separate conditional quasi-Poisson regressions with distributed lag non-linear models including lag days 0 to 21.

Age and sex		Relative risk (95%-CI)
Male		
	<50 years	1.05 (1.04;1.06)
		1.16 (1.06;1.26)
		1.01 (0.95;1.07)
	50-59 years	1.04 (1.02;1.06)
		1.07 (0.91;1.27)
		1.01 (0.97;1.05)
	60-69 years	1.03 (1.02;1.05)
		0.93 (0.77;1.12)
		1.02 (0.99;1.04)
	70-79 years	1.03 (1.02;1.05)
		1.01 (0.82;1.24)
		0.99 (0.97;1.01)
	>79 years	1.05 (1.03;1.06)
		1.20 (0.89;1.62)
		1.04 (1.02;1.07)
	all	1.04 (1.03;1.05)
		1.10 (1.03;1.17)
		1.01 (1.00;1.03)
Female		
	<50 years	1.04 (1.03;1.05)
		1.10 (1.00;1.20)
		0.91 (0.85;0.98)
	50-59 years	1.06 (1.04;1.08)
		1.07 (0.87;1.32)
		1.01 (0.96;1.06)
	60-69 years	1.04 (1.02;1.06)
		1.31 (1.04;1.64)
		1.04 (1.01;1.08)
	70-79 years	1.03 (1.02;1.05)
		0.87 (0.70;1.08)
		1.02 (1.00;1.05)
	>79 years	1.10 (1.08;1.11)
		1.05 (0.83;1.33)
		1.08 (1.06;1.10)
	all	1.05 (1.04;1.06)
		1.08 (1.01;1.16)
		1.05 (1.03;1.06)

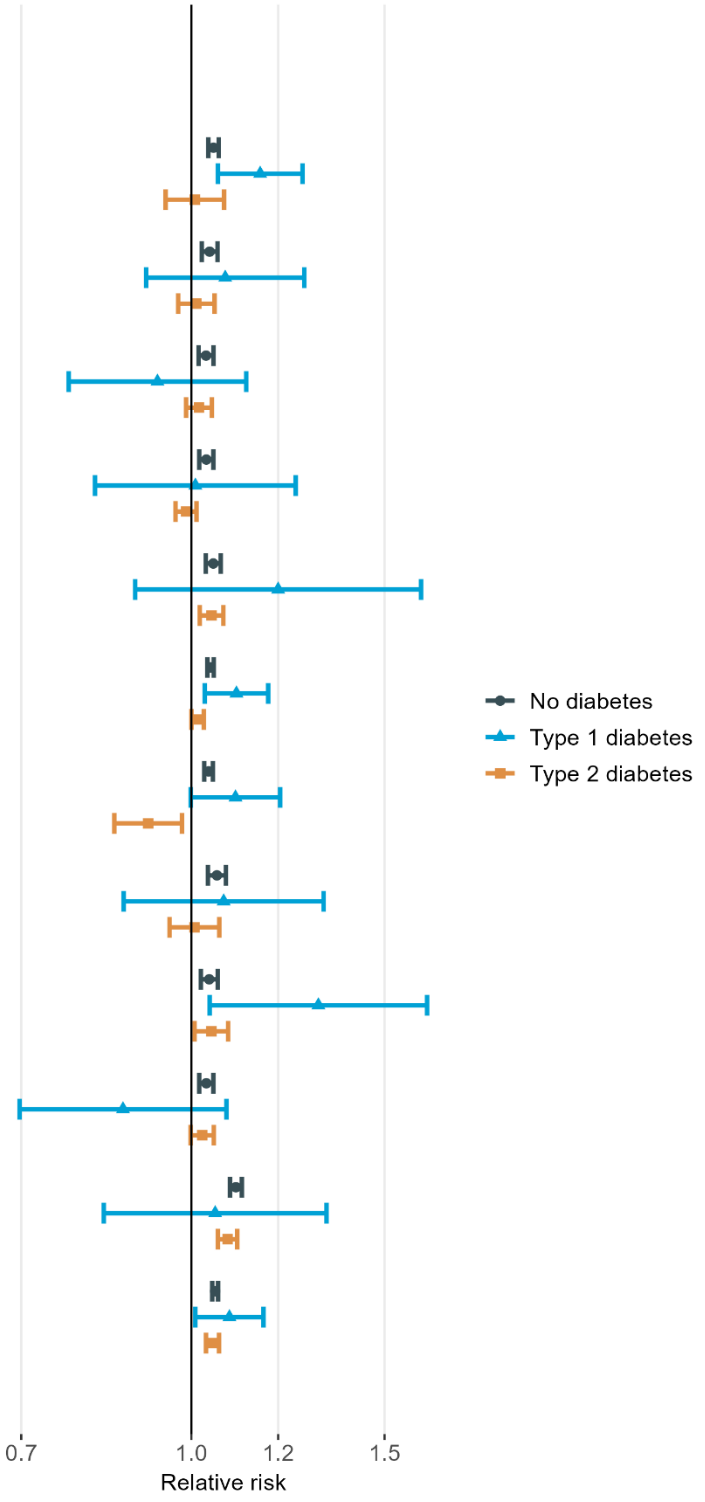

Figure S19. Overall cumulative association between extreme heat and all-cause emergency hospital admission by age, sex and diabetes status – analysis restricted to non-external causes for hospital admission.

Age and sex	Relative risk (95%-CI)
Male	
	0.91 (0.90;0.92)
<50 years	0.96 (0.87;1.05)
	0.94 (0.88;1.00)
	0.93 (0.92;0.95)
50-59 years	0.86 (0.72;1.03)
	0.94 (0.90;0.97)
	1.01 (0.99;1.02)
60-69 years	1.09 (0.90;1.33)
	1.03 (1.00;1.05)
	1.07 (1.06;1.09)
70-79 years	1.22 (0.98;1.51)
	1.06 (1.04;1.09)
	1.11 (1.09;1.13)
>79 years	1.49 (1.08;2.05)
	1.07 (1.05;1.10)
	0.99 (0.98;1.00)
all	1.00 (0.93;1.07)
	1.04 (1.02;1.05)
Female	
	0.93 (0.93;0.94)
<50 years	0.82 (0.74;0.90)
	0.81 (0.75;0.88)
	0.95 (0.93;0.97)
50-59 years	0.98 (0.78;1.23)
	0.98 (0.93;1.03)
	1.00 (0.99;1.02)
60-69 years	1.12 (0.89;1.42)
	1.06 (1.03;1.10)
	1.00 (0.99;1.02)
70-79 years	0.85 (0.68;1.05)
	1.04 (1.01;1.06)
	1.08 (1.06;1.09)
>79 years	1.03 (0.81;1.32)
	1.09 (1.07;1.11)
	0.98 (0.98;0.99)
all	0.89 (0.82;0.96)
	1.05 (1.04;1.06)

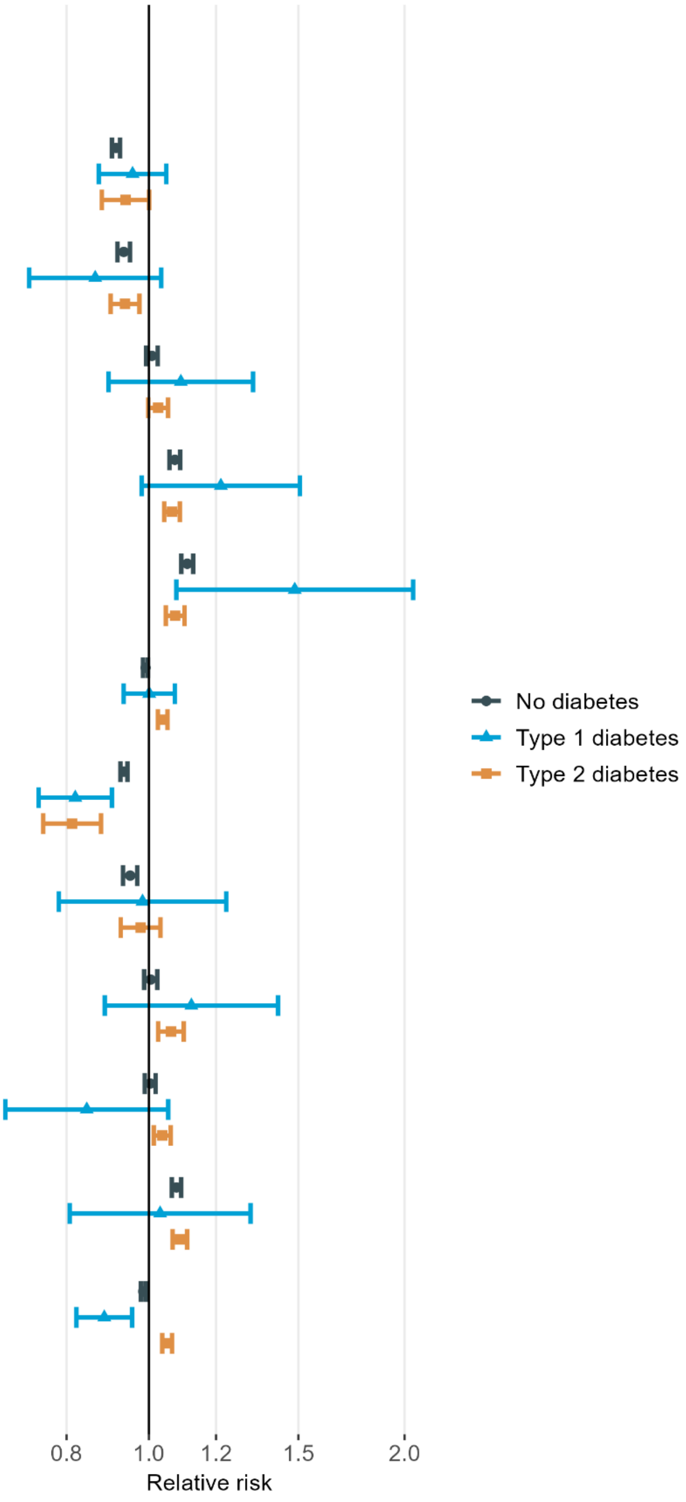

Figure S20. Overall cumulative association between extreme cold and all-cause emergency hospital admission by age, sex and diabetes status – analysis restricted to non-external causes for hospital admission.

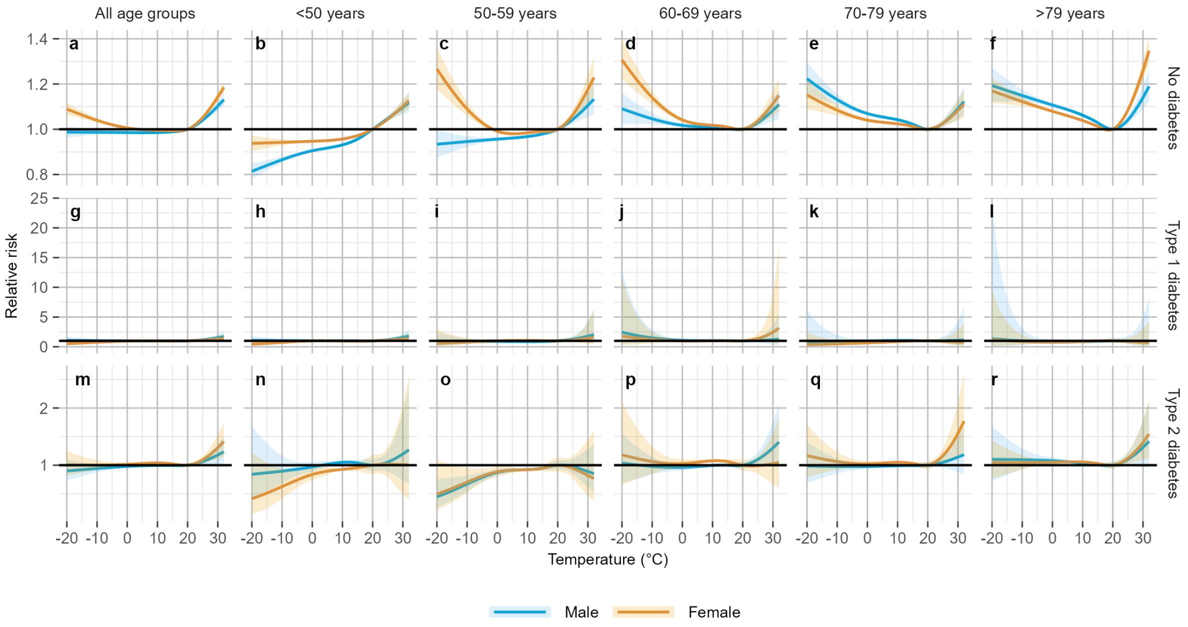

Figure S21. Overall cumulative association between daily air temperature and all-cause emergency hospital admission by age, sex and diabetes status – analysis restricted to cause-specific diabetes hospital admissions. The panels show results for people without diabetes (a-f), with type 1 diabetes (g-l), type 2 diabetes (m-r) including all age groups (a, g, m), age < 50 years (b, h, n), 50-59 years (c, i, o), 60-69 years (d, j, p), 70-79 years (e, k, q) and > 79 years (f, l, r). Estimates are based on separate conditional quasi-Poisson regressions with distributed lag non-linear models including lag days 0 to 21.

Age and sex	Relative risk (95%-CI)
Male	
	1.04 (1.03;1.05)
<50 years	1.15 (1.03;1.29)
	1.04 (0.90;1.22)
	1.04 (1.02;1.05)
50-59 years	1.24 (0.95;1.64)
	0.98 (0.87;1.10)
	1.02 (1.01;1.04)
60-69 years	1.05 (0.74;1.49)
	1.09 (0.99;1.19)
	1.02 (1.01;1.04)
70-79 years	1.02 (0.66;1.57)
	1.04 (0.96;1.14)
	1.03 (1.02;1.05)
>79 years	0.95 (0.52;1.72)
	1.09 (0.99;1.20)
	1.03 (1.03;1.04)
all	1.15 (1.04;1.26)
	1.05 (1.01;1.10)
Female	
	1.04 (1.03;1.05)
<50 years	1.10 (0.97;1.25)
	1.02 (0.81;1.28)
	1.05 (1.04;1.07)
50-59 years	1.11 (0.79;1.56)
	0.96 (0.80;1.14)
	1.03 (1.02;1.05)
60-69 years	1.32 (0.87;2.01)
	0.99 (0.86;1.13)
	1.02 (1.01;1.04)
70-79 years	0.93 (0.62;1.40)
	1.14 (1.04;1.25)
	1.07 (1.06;1.08)
>79 years	0.91 (0.57;1.44)
	1.10 (1.02;1.19)
	1.05 (1.04;1.05)
all	1.10 (0.98;1.22)
	1.08 (1.03;1.14)

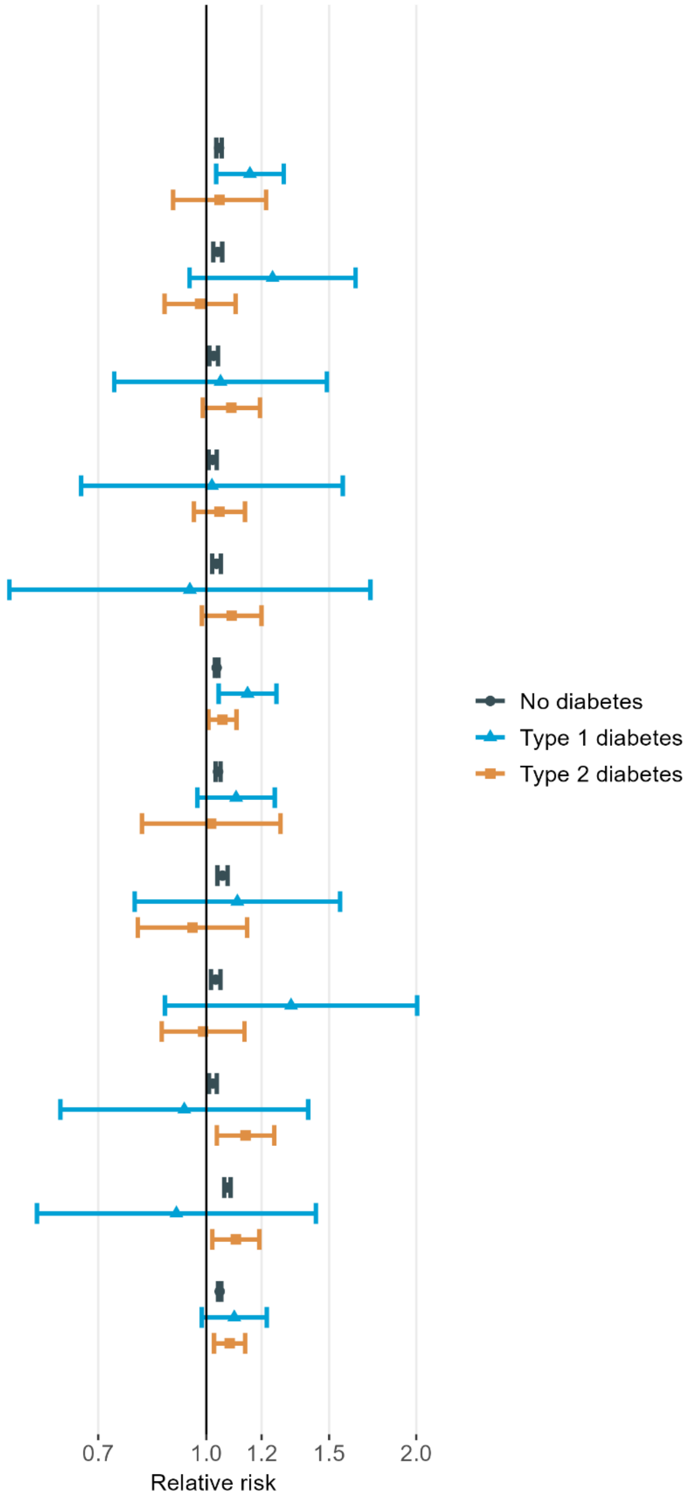

Figure S22. Overall cumulative association between extreme heat and all-cause emergency hospital admission by age, sex and diabetes status – analysis restricted to cause-specific diabetes hospital admissions.

Age and sex	Relative risk (95%-CI)
Male	
	0.88 (0.87;0.89)
<50 years	1.03 (0.92;1.16)
	0.92 (0.78;1.08)
	0.95 (0.94;0.96)
50-59 years	0.86 (0.64;1.17)
	0.75 (0.67;0.85)
	1.03 (1.02;1.05)
60-69 years	1.30 (0.88;1.93)
	0.97 (0.88;1.06)
	1.10 (1.09;1.12)
70-79 years	0.92 (0.56;1.50)
	0.98 (0.90;1.06)
	1.13 (1.12;1.15)
>79 years	0.92 (0.46;1.84)
	1.09 (0.99;1.21)
	0.99 (0.98;0.99)
all	1.02 (0.92;1.12)
	0.96 (0.91;1.00)
Female	
	0.94 (0.94;0.95)
<50 years	0.85 (0.75;0.97)
	0.71 (0.55;0.91)
	1.04 (1.03;1.06)
50-59 years	0.87 (0.60;1.27)
	0.78 (0.65;0.94)
	1.10 (1.08;1.11)
60-69 years	1.10 (0.70;1.73)
	1.04 (0.91;1.19)
	1.07 (1.05;1.08)
70-79 years	0.55 (0.35;0.86)
	1.04 (0.95;1.14)
	1.11 (1.09;1.12)
>79 years	0.82 (0.49;1.35)
	1.04 (0.97;1.12)
	1.03 (1.02;1.03)
all	0.84 (0.75;0.94)
	1.00 (0.96;1.05)

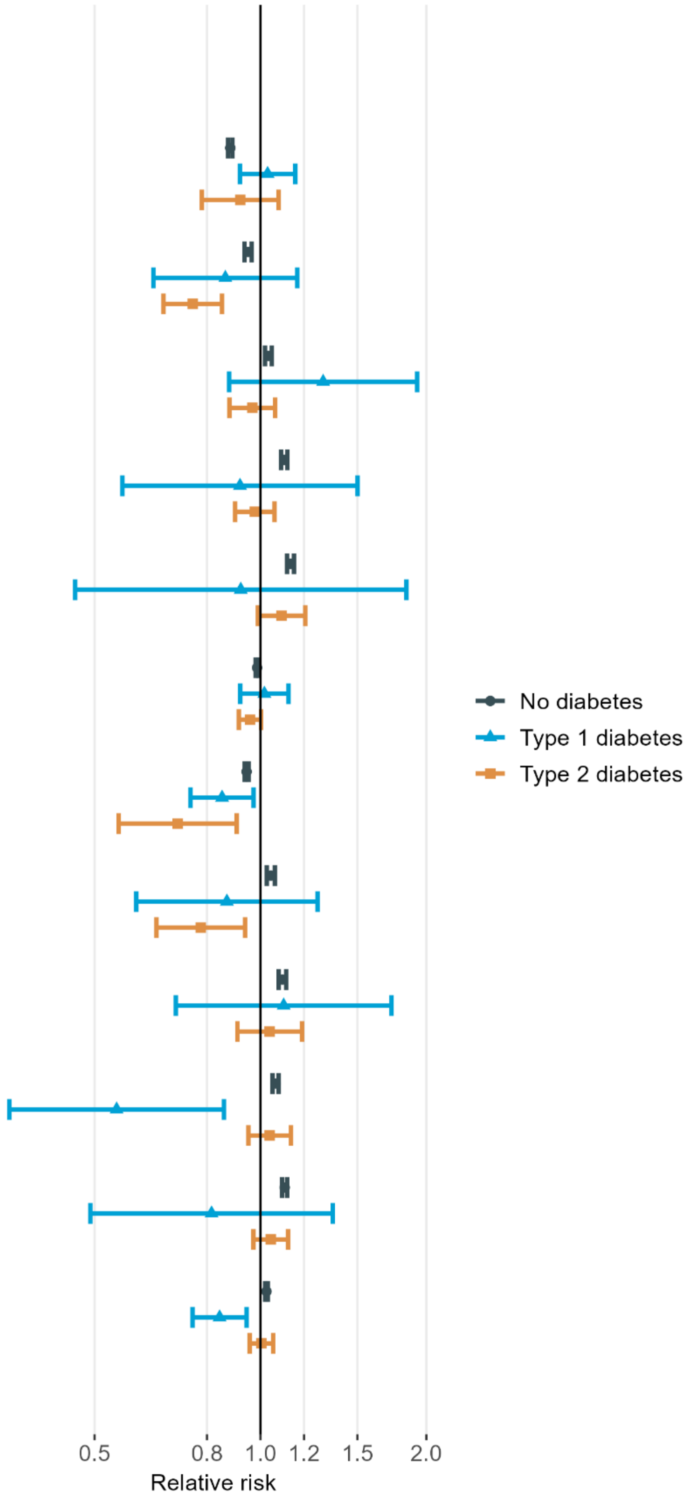

Figure S23. Overall cumulative association between extreme cold and all-cause emergency hospital admission by age, sex and diabetes status – analysis restricted to cause-specific diabetes hospital admissions.
